# Supplementary material for: Safetxt: a safer sex intervention delivered by mobile phone messaging on sexually transmitted infections (STI) among young people in the UK - protocol for a randomised controlled trial
Source: BMJ Open. 2020 Mar 8;10(3):e031635. doi: 10.1136/bmjopen-2019-031635 (PMC7064138; doi:10.1136/bmjopen-2019-031635)
Supplement: Supplementary data [file bmjopen-2019-031635supp001.pdf]

A randomised controlled trial of a safer sex intervention delivered through mobile phone messaging

Local site logo

Participant's Information Sheet
safetxt: a randomised controlled trial of a safer sex intervention

We are inviting you to take part in a research study. Before you decide, it is important that you know why we are doing the study and what is involved. Please read the following information carefully.

What is the study?
This study is testing whether text messages providing information and tips to increase safer sex helps young people adopt safer sex behaviours.

Why have I been chosen?
You are aged 16-24 and have recently received a positive test result for Chlamydia, gonorrhoea or had a non specific urethritis diagnosis.

Do I have to take part?
No, it is up to you to decide whether to take part.

What will happen if I take part?
After you have had all your questions answered and have agreed to take part, we will ask you to complete a consent form. You can do this on our secure and confidential study website, by filling out a paper version, by text message or by email. We will then ask you to complete a short confidential questionnaire providing details about yourself.

After you have completed the questionnaire, **an automated computer system will put you into one of two groups by chance (randomly)**. One group will receive safer sex text messages for 1-2 weeks, then about 5 messages a month for 11 months. The other group will receive text messages asking you to confirm your contact details. These messages will not be about sexual health. This group will receive one message a month for 12 months.

We will ask you to complete the questionnaire again at 1 and 12 months after signing up. This is to see how things have been for you after joining the study. Also at 12 months we will ask you to provide a sample to test for Chlamydia and gonorrhoea. The testing kit will be sent to you by post. **Participating in the study involves returning one urine sample or self-taken swab to test for Chlamydia and gonorrhoea.**

Will you compensate me for the time this takes?
We will offer you £5 as compensation for your time at each follow up: for the 1 month questionnaire and the 12 month questionnaire. You will be compensated for your time for returning your 12 month sample.

We do not charge for text messages we send to you.

What are the alternatives?
You do not have to take part.

What are the possible disadvantages in taking part?
Completing the questionnaires and providing a sample will take up some of your time. It is possible that the messages we send could be read by someone else. If you are concerned about this, you can password-lock your phone and delete the messages after you read them.

Free C, *et al*. *BMJ Open* 2020; 10:e031635. doi: 10.1136/bmjopen-2019-031635

What are the possible benefits of taking part?
We cannot promise that your health will benefit from taking part but you may find the messages helpful and you may learn about safer sex behaviours.

What happens when this study stops?
When the study stops, we will look at the data collected to see if the text messages helped increase safer sex behaviours.

What will happen if I don't want to carry on with the study?
You can stop receiving text messages by texting STOP or pause the text messages by texting PAUSE. You can withdraw at any time by letting the Clinical Trials Unit know (safetxt@LSHTM.ac.uk or freephone 0800 008 7197). You do not have to give a reason for wanting to withdraw.

What if there is a problem?
You can contact the Clinical Trials Unit if there is a problem. If you would like to make a formal complaint, contact Dr Caroline Free who will follow the complaints procedure.

Will my taking part in this study be kept confidential?
Yes. Your answers to the questionnaires will be stored anonymously and your contact details and Chlamydia and gonorrhoea test will be kept confidential. You will be allocated a unique study number when you enrol in the study. We will not inform your parents about your involvement in this research. If you were tested for sexually transmitted infections during the study, we will ask your clinic or GP for your test results. We will also ask for information on whether or not a partner was treated. To keep the messages confidential, password-lock your phone and delete messages after you read them.

Your records will be made available to people authorised to work on the study. They may also need to be made available to people for ensuring that the study is carried our correctly.

With your permission, we will ask your clinic or GP for your test results for sexually transmitted infections after you have finished participating in the study. This is to allow the study researchers to look into long term effects about your sexual health.

What will happen to the samples that I give?
You will post your sample directly to a laboratory to test for Chlamydia and gonorrhoea infection. The laboratory will destroy the samples after they have finished testing. You will be notified if the test shows that there is Chlamydia or gonorrhoea infection. The clinic where you signed up to the study will contact you to arrange treatment.

How long will your data be stored for?
Your data will be kept for 10 years after the trial is completed. However, in accordance with the Data Protection Act of 1998, any personal identifiable data will not be kept longer than necessary and will be deleted within 3 months after you have finished the study.

What will happen to the results of the research study?
The results will be published in a scientific journal so that other people know about it. If you would like a copy of the results please contact the Clinical Trials Unit.

If you want to receive the safer sex messages after your participation in the study, please let us know and we will send them to you.

If the results of the study show that the messages have helped, they will be made available to all young people.

Who is organising and funding the research?
The study is run by Dr Caroline Free (Chief Investigator) from the London School of Hygiene and Tropical Medicine, at the University of London. The UK National Institute for Health Research is funding the trial.

Free C, *et al. BMJ Open* 2020; 10:e031635. doi: 10.1136/bmjopen-2019-031635

Who has reviewed the trial?

London Riverside NHS Medical Research Ethics Committee.

Thank you for taking the time to consider taking part.

If you would like further information please contact the local Research Nurse or Clinical Trials Unit (details below)

The team contact details:

INSERT LOCAL CONTACT DETAILS

If you have any questions about the trial, please email: safetxt@LSHTM.ac.uk or Freephone: 0800 008 7197

Free C, *et al. BMJ Open* 2020; 10:e031635. doi: 10.1136/bmjopen-2019-031635
