# Supplementary material for: Safetxt: a safer sex intervention delivered by mobile phone messaging on sexually transmitted infections (STI) among young people in the UK - protocol for a randomised controlled trial
Source: BMJ Open. 2020 Mar 8;10(3):e031635. doi: 10.1136/bmjopen-2019-031635 (PMC7064138; doi:10.1136/bmjopen-2019-031635)
Supplement: Supplementary data [file bmjopen-2019-031635supp002.pdf]

safetxt
A randomised controlled trial of a safer sex intervention delivered through mobile phone messaging

Hospital logo to be inserted

Consent Form

Chief Investigator: Dr Caroline Free

Name of local researcher: *(Please insert local details)*

Please **initial box**

I confirm that I have read and understand the information sheet [version 8, 16th April 2018] for the above study. I have had the opportunity to consider the information, ask questions and have had these answered satisfactorily. ☐

I understand that participation in this study is my choice and that I am free to withdraw at any time without giving any reason, without my medical care or legal rights being affected. ☐

I understand that I should not read or send text messages while driving a vehicle. ☐

I understand that relevant sections of data collected during the study may be looked at by the NHS Trust or LSHTM, where it is relevant to my taking part in this research. I give permission for these individuals to have access to my records. ☐

I agree to the study researchers letting my clinic know of any positive test results I receive so they can arrange treatment. ☐

I agree that my clinic/GP can provide the researchers with my test results for sexually transmitted infections. ☐

If I give my partner(s) details to my clinic, I agree to the researchers being told if they have been tested. ☐

I understand that all information I provide will remain confidential in accordance with the Data Protection Act of 1998. ☐

I understand that the information collected about me will be used to support other research in the future, and may be shared anonymously with other researchers. ☐

I agree to take part in the above study. ☐

Optional

After the study, I agree that my clinic/GP can provide the researchers with my sexually transmitted infection results. ☐

Name of Patient	Date and time	Signature
Name of researcher taking consent	Date and time	Signature

Consent Form Version 8 16th May 2018 ISRCTN64390461
