# Supplementary material for: Safetxt: a safer sex intervention delivered by mobile phone messaging on sexually transmitted infections (STI) among young people in the UK - protocol for a randomised controlled trial
Source: BMJ Open. 2020 Mar 8;10(3):e031635. doi: 10.1136/bmjopen-2019-031635 (PMC7064138; doi:10.1136/bmjopen-2019-031635)
Supplement: Author's manuscript [file bmjopen-2019-031635.draft_revisions.pdf]

BMJ Open is committed to open peer review. As part of this commitment we make the peer review history of every article we publish publicly available.

When an article is published we post the peer reviewers' comments and the authors' responses online. We also post the versions of the paper that were used during peer review. These are the versions that the peer review comments apply to.

The versions of the paper that follow are the versions that were submitted during the peer review process. They are not the versions of record or the final published versions. They should not be cited or distributed as the published version of this manuscript.

BMJ Open is an open access journal and the full, final, typeset and author-corrected version of record of the manuscript is available on our site with no access controls, subscription charges or pay-per-view fees (http://bmjopen.bmj.com).

If you have any questions on BMJ Open's open peer review process please email info.bmjopen@bmj.com

BMJ Open

safetxt: a safer sex intervention delivered by mobile phone messaging on sexually transmitted infections (STI) among young people in the UK- protocol for a randomised controlled trial

Journal:	*BMJ Open*
Manuscript ID	bmjopen-2019-031635
Article Type:	Protocol
Date Submitted by the Author:	14-May-2019

Complete List of Authors:	Free, Caroline; London School of Hygiene and Tropical Medicine, Population Health<br>McCarthy, Ona; London School of Hygiene and Tropical Medicine, Population Health<br>Palmer, Melissa; London School of Hygiene and Tropical Medicine, Population Health<br>Knight, Rosemary; London School of Hygiene and Tropical Medicine, Clinical Trials Unit, MSD<br>Edwards, Phil; London School of Hygiene and Tropical Medicine, Population Health<br>French, Rebecca ; London School of Hygiene and Tropical Medicine, Social and Environmental Health Research<br>Baraitser, Paula; King's College London, Centre for Global Health<br>Hickson, Ford; London School of Hygiene and Tropical Medicine, Sigma Research<br>Wellings, Kaye; London School of Hygiene and Tropical Medicine, Social and Environmental Health Research<br>Roberts, Ian; London School of Hygiene and Tropical Medicine, Population Health<br>Bailey, Julia; University College London, Primary Care and Population Health<br>Hart, Graham; University College London, Department of Infection and Population Health<br>Michie, Susan; University College London, Centre for Outcomes Research and Effectivenes<br>Clayton, Tim; London School of Hygiene and Tropical Medicine<br>Ploubidis, George; University College London Institute of Education<br>Carpenter, James; London School of Hygiene and Tropical Medicine<br>Turner, Katy; University of Bristol<br>Devries, Karen; London School of Hygiene and Tropical Medicine<br>Potter, Kimberley; London School of Hygiene and Tropical Medicine

Keywords:	sexually transmitted infection, sexual behaviour, text message, mHealth, randomised controlled trial, young people

SCHOLARONE™
Manuscripts

I, the Submitting Author has the right to grant and does grant on behalf of all authors of the Work (as defined in the below author licence), an exclusive licence and/or a non-exclusive licence for contributions from authors who are: i) UK Crown employees; ii) where BMJ has agreed a CC-BY licence shall apply, and/or iii) in accordance with the terms applicable for US Federal Government officers or employees acting as part of their official duties; on a worldwide, perpetual, irrevocable, royalty-free basis to BMJ Publishing Group Ltd ("BMJ") its licensees and where the relevant Journal is co-owned by BMJ to the co-owners of the Journal, to publish the Work in this journal and any other BMJ products and to exploit all rights, as set out in our licence.

The Submitting Author accepts and understands that any supply made under these terms is made by BMJ to the Submitting Author unless you are acting as an employee on behalf of your employer or a postgraduate student of an affiliated institution which is paying any applicable article publishing charge ("APC") for Open Access articles. Where the Submitting Author wishes to make the Work available on an Open Access basis (and intends to pay the relevant APC), the terms of reuse of such Open Access shall be governed by a Creative Commons licence – details of these licences and which Creative Commons licence will apply to this Work are set out in our licence referred to above.

Other than as permitted in any relevant BMJ Author's Self Archiving Policies, I confirm this Work has not been accepted for publication elsewhere, is not being considered for publication elsewhere and does not duplicate material already published. I confirm all authors consent to publication of this Work and authorise the granting of this licence.

For peer review only

Title Page

safetxt: a safer sex intervention delivered by mobile phone messaging on sexually transmitted infections (STI) among young people in the UK- protocol for a randomised controlled trial.

Authors

Caroline Free, Department of Population Health, Faculty of Epidemiology and Population Health, London School of Hygiene & Tropical Medicine, Keppel Street, London, WC1E 7HT. caroline.free@lshtm.ac.uk

Ona L McCarthy, Department of Population Health, Faculty of Epidemiology and Population Health, London School of Hygiene & Tropical Medicine, Keppel Street, London, WC1E 7HT. ona.mccarthy@lshtm.ac.uk

Melissa Palmer, Department of Population Health, Faculty of Epidemiology and Population Health, London School of Hygiene & Tropical Medicine, Keppel Street, London, WC1E 7HT. melissa.palmer@lshtm.ac.uk

Rosemary Knight, Clinical Trials Unit. Department of Medical Statistics, Faculty of Epidemiology and Population Health, London School of Hygiene & Tropical Medicine, Keppel Street, London, WC1E 7HT. rosemary.knight@lshtm.ac.uk

Phil Edwards, Department of Population Health, Faculty of Epidemiology and Population Health, London School of Hygiene & Tropical Medicine, Keppel Street, London, WC1E 7HT. phil.edwards@lshtm.ac.uk

Rebecca French, Department of Public Health Environments and Society, Faculty of Public Health and Policy, London School of Hygiene & Tropical Medicine, 15-17 Tavistock Place, London, WC1H 9SH. rebecca.french@lshtm.ac.uk

Paul Baraitser, King's Centre for Global Health, King's College London, Camberwell Sexual Health Centre, 100 Denmark Hill, London, SE5 9RS. paula_baraitser@mac.com

Ford Hickson, Department of Social and Environmental Health Research, Faculty of Public Health and Policy, London School of Hygiene & Tropical Medicine, 15-17 Tavistock Place, London, WC1H 9SH. ford.hickson@lshtm.ac.uk

Kaye Wellings, Department of Public health Environments and Society, Faculty of Public Health and Policy, London School of Hygiene & Tropical Medicine, 15-17 Tavistock Place, London, WC1H 9SH. kaye.wellings@lshtm.ac.uk

For peer review only - http://bmjopen.bmj.com/site/about/guidelines.xhtml

Ian Roberts, Department of Population Health, Faculty of Epidemiology and Population Health, London School of Hygiene & Tropical Medicine, Keppel Street, London, WC1E 7HT. ian.roberts@ucl.ac.uk

Julia V Bailey, Department of Primary Care and Population Health, University College London, Gower Street, London, WC1E 6BT. julia.bailey@ucl.ac.uk

Graham Hart, Faculty of Population Health Sciences, University College London, Gower Street, London, WC1E 6BT. g.hart@ucl.ac.uk

Susan Michie, Department of Clinical, Educational and Health Psychology, University College London, Gower Street, London, WC1E 6BT. s.michie@ucl.ac.uk

Tim Clayton. Department of Medical Statistics. Faculty of Epidemiology and Population Health, London School of Hygiene & Tropical Medicine, Keppel Street, London, WC1E 7HT. Tim.clayton@lshtm.ac.uk

George Ploubidis, Department of Social Science, Institute of Education, University College London, Gower Street, London, WC1E 6BT. g.ploubidis@ucl.ac.uk

James Carpenter, Department of Medical Statistics. Faculty of Epidemiology and Population Health, London School of Hygiene & Tropical Medicine, Keppel Street, London, WC1E 7HT. james.carpenter@lshtm.ac.uk

Katy Turner, Bristol Veterinary School, University of Bristol, Churchill Building, Langford Campus, Bristol, BS40 5DU katy.turner@bristol.ac.uk

Karen Devries, Faculty of Public Health and Policy. LSHTM, 15-17 Tavistock Place, London WC1H 9SH. karen.devries@lshtm.ac.uk

Kimberley Potter, Clinical Trials Unit. Department of Medical Statistics, Faculty of Epidemiology and Population Health, London School of Hygiene & Tropical Medicine, Keppel Street, London, WC1E 7HT. kimberley.potter@lshtm.ac.uk

For peer review only - http://bmjopen.bmj.com/site/about/guidelines.xhtml

Abstract

Introduction

Young people aged 16-24 have the highest prevalence of genital chlamydia and gonorrhoea compared to other age groups and re-infection rates following treatment are high. Long-term adverse health effects include sub-fertility and ectopic pregnancy, particularly among those with repeated infections. We developed the safetxt intervention delivered by text message to reduce sexually transmitted infection (STI) by increasing partner notification, condom use and (STI) testing among young people in the United Kingdom (UK).

Methods and analysis

A single blind randomised trial to reliably establish the effect of the safetxt intervention on chlamydia and gonorrhoea infection at one year. We will recruit 6250 people aged 16-24 years who have recently been diagnosed with chlamydia, gonorrhoea or non-specific urethritis from health services in the UK. Participants will be allocated to receive the safetxt intervention (text messages designed to promote safer sexual health behaviours) or to receive the control text messages (monthly messages asking participants about changes in contact details) by an automated remote online randomisation system.

The primary outcome will be the cumulative incidence of chlamydia and gonorrhoea infection at one year assessed by nucleic acid amplification tests. Secondary outcomes include partner notification, correct treatment of infection, condom use, and STI testing prior to sex with new partners.

Ethics and dissemination

Ethics approval was obtained from NHS Health Research Authority - London – Riverside Research Ethics Committee (REC reference: 15/LO/1665) and the LSHTM. We will submit the results of the trial for publication in peer reviewed journals.

Trial registration

International Standard Randomised Controlled Trials Number: ISRCTN64390461. Registered on 17th March 2016. *World Health Organization trial registration dataset*

Available at: http://apps.who.int/trialsearch/Trial2.aspx?TrialID=ISRCTN64390461

Trial Protocol version: 12 19th July 2018

For peer review only - http://bmjopen.bmj.com/site/about/guidelines.xhtml

Strengths and limitations of this study

- The study design is a large randomised trial to assess the effect of 'safetxt', a safer sex intervention delivered by text message, on objectively measured STI (chlamydia and gonorrhoea) infection at one year.
- The use of an independent remote computer-based randomisation system linked to an automated message delivery system ensures allocation concealment.
- The secondary outcomes will assess the effect of the intervention on safer sex behaviours: partner notification, condom use and testing before sex with a new partner.
- The process evaluation will explore which intervention components are effective and has the potential to generate general principles to inform similar interventions in the future.
- The inability to blind participants receiving a behavioural intervention is a limitation of the trial, which could introduce bias in the ascertainment of self-reported outcomes.

Keywords

Sexually transmitted infection (STI); sexual behaviour; young people; mHealth; randomised controlled trial, text message.

For peer review only - http://bmjopen.bmj.com/site/about/guidelines.xhtml

INTRODUCTION

Younger people aged 16-24 bear the heaviest burden of sexually transmitted infections (STIs) such as chlamydia and gonorrhoea, and their long-term adverse health effects including ectopic pregnancy and subfertility (1, 2). The risk of adverse health effects increases with repeated infections, and re-infection rates following treatment are high: up to 30% for chlamydia and 12% for gonorrhoea at one year (3). Those with an STI are more likely to acquire further STIs and HIV, if exposed. There are marked inequalities in sexual health; STIs are positively associated with lower educational level (4) and living in more deprived areas (1, 5, 6).

Partner notification, condom use and STI testing can reduce infection and re-infection. There is some evidence that existing interventions delivered face-to-face that target condom use and/or STI testing may be effective, but they are limited in their reach or too costly for widespread application (7). Existing interventions delivered via the media have high reach but their effects have yet to be established (8). Effective ways to increase partner notification in specialist and primary care settings are needed (9, 10).

Mobile phones have the potential to provide effective, low cost health behaviour support (11, 12). However, evidence for the effect of mobile phone support for safer sex behaviours such as condom use, partner notification and STI testing is equivocal (13-15). Interventions have targeted testing for STI (16-21), delaying resumption of sexual activity until 42 days after circumcision (22) and condom use (17, 18, 23, 24), but none have aimed to increase partner notification. The effect of mobile phone based interventions on STI is not known.

In the UK, 99% of 16-24 year olds are mobile phone users (ONS, 2012) and mobile phone ownership is high across all socio-economic groups. In research leading to this trial, it was demonstrated that interactive support via text message is particularly acceptable in the area of sexual health intervention (15, 25, 26).

The safetxt trial builds on the successful intervention development work and pilot trial (15, 26, 27). To develop the intervention, we convened a working group including experts in health psychology, contraception research, contraception service provision, IT and the development of interventions delivered by mobile phone. The intervention messages were developed based on: behaviour change theory; evidence-based behaviour change techniques; the content of effective face-to-face safer sex interventions; the factors known to influence safer sex behaviours; the views of 82 young people collected in focus groups and a questionnaire completed by 100 people aged 16-24 (15). The theory and evidence-based intervention is designed to reduce STIs in young

For peer review only - http://bmjopen.bmj.com/site/about/guidelines.xhtml

people by supporting them in telling a partner about an infection, using condoms and obtaining testing before unprotected sex with a new partner. In a qualitative study with young people, recipients reported that the tone, language, content, and frequency of messages was appropriate (26). Messages reportedly increased knowledge and confidence in how to use condoms and reduced stigma enabling them to tell a partner about an STI. Sharing messages with their partner enabled participants to negotiate condom use.

The pilot trial demonstrated that methods of recruitment, randomisation, intervention delivery and follow-up were successful and that a full-scale randomised controlled trial of the safetxt intervention is feasible (27).

Objectives

The primary objective of this trial is to quantify the effects of the safetxt intervention compared to a control group receiving usual care and messages about trial participation on chlamydia or gonorrhoea infection at one year. Secondary objectives are to determine the effects of safetxt on partner notification and condom use at 4 weeks and on condom use and STI testing at one year. Which intervention components are effective will be explored by collecting data on the theoretical constructs on the pathway to behaviour change influenced by the intervention components. The cost-effectiveness of the intervention will be established.

METHODS AND ANALYSIS

Design and setting

Safetxt is a single blind parallel group randomised superiority trial with a 1:1 allocation ratio designed to establish the effects of a safer sex intervention delivered by text message on the cumulative incidence of Chlamydia and gonorrhoea infection at one year. (See Figure 1 for the trial flow chart).

Potential participants testing positive for Chlamydia, gonorrhoea or diagnosed with non-specific urethritis (NSU) will be identified from UK STI testing services by research staff based at the service (a full list of participating services is available at http://safetxt.lshtm.ac.uk/participating-sites/). The intervention is delivered by text message in the community.

Characteristics of participants

Inclusion criteria

Participants will be eligible if they are between 16 and 24 years of age, own a personal mobile phone, are able to provide informed consent, and have either been diagnosed with chlamydia,

For peer review only - http://bmjopen.bmj.com/site/about/guidelines.xhtml

For peer review only

gonorrhoea, or NSU in the last 2 weeks, or have started treatment for Chlamydia, gonorrhoea or NSU in the last two weeks.

Exclusion criteria

Participants will be excluded if they are known to be a sexual partner of someone already recruited to the trial.

Recruitment and consent

Research staff based at STI testing services will identify potential participants. They will provide potential participants with trial information at one of three time points, (i) when potential participants attend the service and are diagnosed with Chlamydia, gonorrhoea or NSU; (ii) receive positive test results for Chlamydia or gonorrhoea by phone; or (iii) collect treatment for Chlamydia, gonorrhoea or NSU from services.

Research staff will provide potential participants with verbal and written information (Supplementary file 1) about the trial and/or participants will view trial information on the website. Participants will be able to join the trial either by, providing informed written consent to the research staff, or by providing consent online (Supplementary file 2) at the trial website or site staff can ask eligible participants if they are happy for their details to be given to research assistants based at the London School of Hygiene & Tropical Medicine Clinical Trials Unit (LSHTM CTU). In this case, LSHTM CTU will contact participants to recruit to the trial. Strategies for achieving adequate enrolment include site visits, training, feedback and rewards, sharing successful recruiting strategies and newsletters.

Allocation and blinding

Participants will be randomly allocated (1:1 ratio), using a computer-based randomisation system, to a safer sex intervention delivered by text messaging, or to a control group, ensuring allocation concealment. Allocation concealment is ensured as the randomisation system is independent, automated and remote.

An electronic link to the computer-based randomisation programme will result in the generation of a research number and allocation to the intervention or control group. The system will then automatically deliver intervention or control group texts according to the allocation.

Given the nature of the intervention, participants will be aware of their assigned group. Research assistants entering data, laboratory staff and researchers conducting the final analysis are masked to allocation until after the analysis is complete.

For peer review only - http://bmjopen.bmj.com/site/about/guidelines.xhtml

Interventions:

Safetxt Intervention group

The intervention aims to increase safer sex in three ways: i) encouraging participants to correctly follow STI treatment instructions including informing partner(s) about infection; ii) promoting condom use with new or casual partners; and iii) encouraging participants to obtain testing for STI prior to unprotected sex. Participants in the intervention group will receive regular messages delivered by text message in community settings according to a predetermined schedule. The intervention was informed by the capability, opportunity and motivation model of behaviour (COM-B)(28). It aims to influence the knowledge, beliefs, self-efficacy, skills, social and interpersonal influences that have important effects on motivation, capability and opportunity to reduce sexual risk behaviour.

Over the first ten days participants are sent messages targeting engagement with the intervention, taking treatment, avoiding sex for 7 days after treatment and telling partner (s) about an infection. These messages provide non-judgemental, non-stigmatising information about STI. They provide suggestions about when, where, and how to tell partner(s) about an infection and examples of how others told partners covering a range of different types of relationship (e.g. casual, long term).

Messages then target condom use and testing for STIs before having sex without a condom with a new partner. Topics cover risk assessment, instructions on how to use condoms, positive aspects of condom use, tips on preventing condom problems and examples of how others resolved condom use problems. Participants are prompted to think about their own success in achieving safer sex strategies, risks they had taken and what they could do differently in the future. Messages included advice regarding testing before unprotected sex with a new partner. Participants are sent links to: services; support for those concerned about partner violence; and web-based information regarding contraception, alcohol and sexual risk, how to use a condom and general communication about sex. The messages provide social support for safer sex behaviours and acknowledge participants experiences.

The intervention employs educational, enabling and incentivising behaviour change functions and twelve behaviour change techniques: information about health consequences of behaviour; instruction on how to carry out the behaviour; demonstrations of risk reduction behaviour; social support; emotional support; social rewards; non-specific incentives; encouragement to add objects to the environment; anticipated regret; problem solving; action planning techniques; and

For peer review only - http://bmjopen.bmj.com/site/about/guidelines.xhtml

reframing (29) . The information on safer sexual practices is in accordance with existing guidelines.

The core message sets include: 74 messages for women who have sex with men (WSM) and women who had sex with men and women (WSMW); 42 messages for women who have sex only with women (WSW); 79 messages for men who have sex with women (MSW) and men who have sex with men and women (MSMW); and 76 messages for men who have sex with men (MSM). Recipients can request additional messages on specific topics. Participants are sent: 4 messages per day for days 1-3, then 1-2 messages per day for days 4-28, then 2-3 messages per week for month 2, and 2-5 messages per month for months 3-12

The messages are tailored according to gender and sexual orientation. WSM, MSM, MSMW, WSMW are sent messages covering how others had negotiated condom use. WSM and MSW are sent messages about emergency contraception. MSM and MSMW are sent messages about post exposure prophylaxis (PEP). WSW only are not sent messages about condom use. The information provided is specific to the STI diagnosed.

Control group

Participants in the control group will receive a monthly text message asking for information about changes in postal or email addresses.

All participants will receive usual care and will be free to seek any other existing service or support they wish.

All messages are sent automatically from a large database to an aggregator, which conveys messages to each participant in the community via their network. The success of delivery of messages at each step is monitored by the aggregator and computer system that generates the messages. A member of the trial team will automatically be notified if there is any failure in the delivery of messages. All participants will be able to set embargoed times when they do not want to receive messages. Participants will be able to stop text messages, but continue with the trial follow up, by texting 'stop' to the short code number.

Outcomes

Our primary outcome will be the cumulative incidence of chlamydia and gonorrhoea infection at one year assessed by nucleic acid amplification tests: urine for men (with additional pharyngeal and anal swabs for MSM) and self-taken vulvo-vaginal swab for women.

For peer review only - http://bmjopen.bmj.com/site/about/guidelines.xhtml

Secondary outcomes

Secondary outcomes at four weeks

- Whether participants took the (prescribed antibiotic) treatment and avoided sex for 7 days after treatment
- Whether they told the last person they had sex with before testing positive, that they needed to get treatment
- Clinic attendance by partner for treatment (identified from clinic records)
- Condom use at last sex
- Process outcomes – scores of the theoretical constructs underlying the components of the intervention (behaviour change mediators):
    - Attitudes towards partner notification
    - Self–efficacy in telling a partner about an infection
    - Self–efficacy in negotiating condom use
    - Correct condom use self-efficacy
    - Knowledge related to STIs

Secondary outcomes at one year

- Diagnosed with any STI after joining the trial according to self-report confirmed by postal test results and clinic records
- Condom use at last sex
- Number of sexual partners since joining the trial
- Sex with someone new since joining the trial
- Condom use at first sex with most recent new partner
- Self-reported sexually transmitted infection testing for self - prior to first sex with most recent new partner
- Sexually transmitted infection testing for self - prior to first sex with most recent new partner (confirmed according to clinic record)
- Participant's report as to whether their most recent new partner was tested for sexually transmitted infection prior to sex with them
- Number of text messages read
- Whether anyone else read the messages
- Contamination between intervention and control group
- Car accident where the participant was the driver in the past year
- Experience of partner violence in the past year

For peer review only

For peer review only - http://bmjopen.bmj.com/site/about/guidelines.xhtml

Data collection

The primary outcome will be assessed using Chlamydia and gonorrhoea tests collected by post at one year and clinic records of completed tests. STI test kits for chlamydia and gonorrhoea will be posted (in P650 standard packaging) to respondents. Directions in the pack will ask participants to provide a vaginal swab (women), a urine sample (men), and additional anal and pharyngeal samples (men who have sex with men) and post it in the prepaid and addressed envelope to the laboratory. Test kits will be identified by lab number only, rendering the laboratory staff blind to the participant's allocation. The accredited laboratory will destroy samples after testing is completed.

Results of the STI testing will be reported on the secure trial lab site by lab code only. Clinic records will be checked to identify participants who have had a positive STI diagnosis since joining the study, confirm self-reported STI testing and partner attendance for treatment.

At 4 weeks and at one-year, postal questionnaires will be sent to all participants to collected self-reported data. Non-responders will be contacted by any method the participant agrees to at enrolment (post, email, text message, telephone call). Participants can directly enter self-reported outcome data via a web-based data entry form. Paper based self-reported outcome data or self-reported data collected by telephone will be directly entered into the web-based data entry form by a trial assistant blinded to treatment allocation.

Participants will be sent a £5 unconditional incentive with each postal request i.e. when sending the 4 weeks questionnaire and 1-year test and questionnaire. Participants who return the test sample will be sent £20. See Figure 2 for the Schedule of enrolment, interventions and assessments. All outcome data will be collected on participants who choose to stop messages unless they withdraw from the trial.

Data management

Anonymised research data will be held on a secure system and password protected. All data systems will be set up with checks to alert the trial assistants if data being entered are illogical, inconsistent or incomplete.

Personal details will be stored on a password protected computer held on a secure server at the LSHTM. In accordance with the Data Protection Act of 1998 (30), this information will be stored separately from any anonymised research data, and will be deleted at the end of the study.

For peer review only - http://bmjopen.bmj.com/site/about/guidelines.xhtml

Any paper data will be locked in a cabinet within a room. All trial procedures are in accordance with the principles of Good Clinical Practice. Essential documents of the sponsor/trial organisers and investigators will be retained for at least ten years after completion of the trial.

Sample size

The study is powered for the primary outcome measure of cumulative incidence of chlamydia and/or gonorrhoea infection at one year. Two main factors determine the number of participants needed for this trial: the estimated event rate, and the size of the treatment effect. Our estimates are based on the following data:

Estimated event rate

The estimated event rate for the cumulative incidence of STI at 1 year is 20%, based on the event rate in cohort studies and the pilot trial (3).

Size of treatment effect

Because the intervention can be administered to large populations at low cost, even a modest reduction in treatable STIs would be worthwhile. The trial has therefore been designed to detect a reduction in chlamydia or gonorrhoea infection of 20% versus 16% (RR 0.8), which is similar to the effects of face-to-face safer sex interventions (7).

Sample size calculation

In the pilot trial 2% of the control group viewed messages delivered to intervention participants. If the real difference in STI infection at one year follow up is 20% versus 16% then with contamination of 2% the trial would detect a difference of 19.9% vs 16%. (Calculated based on 2% of the control group having an infection rate the same as the intervention group = (98% x 20%) + (2% x16%) = 19.9%).

Five thousand participants would provide 90% power to detect this difference, allowing for 20% losses to follow-up.

The TSC reviewed the blinded event rate after 546 patients had completed 12 months follow up and recommended an increase in the sample size to 6250 due to a lower than expected event rate of 15.6%.

Analysis

A detailed statistical analysis plan (SAP) will be finalised and submitted to the TSC before the end of the trial and unblinding. For the primary outcome and other binary outcomes, we will

For peer review only - http://bmjopen.bmj.com/site/about/guidelines.xhtml

estimate any differences between the groups using logistic regression and we will report odds ratios with 95% confidence intervals and p-values, adjusting for key baseline predictors of outcome. These will specified in advance in the SAP. For continuous outcomes, we will use linear regression to test for a difference in mean scores between the arms. All analyses will be based on the intention-to-treat principle.

For the primary analysis Multiple Imputation by Chained Equations (MICE) will be used in order to account for missing data (31). MICE makes appropriate assumptions for accommodating missing data in the analysis based on the predictors of outcome and the predictors of loss to follow up. MICE is recognised as a way of reducing bias and increasing precision of trial results and is increasingly used as the primary analysis in randomised controlled trials (12, 31). One hundred imputed datasets will be generated and the point estimates and standard errors will be combined using Rubin's rules. We will perform information-anchored sensitivity analysis using the delta-method (32).

Supplementary analyses.

Before fitting our primary analysis model, we need to identify appropriate auxiliary variables to include in the imputation model. Such auxiliary variables should have information about the missing outcome. Specifically, an independent statistician will use a dataset without the treatment variable and perform regression analyses to identify key predictors of outcomes and which combination of baseline variables and time to negative STI test in clinic should be included as auxiliary variables in the imputation model. We will supplement this by performing and reporting a complete case analysis (where any participants with missing information on any covariate or outcome shall be excluded).

Sub group analyses

A limited number of subgroup analyses will be undertaken adjusting for other key predictors of outcome. Subgroup analyses include: age (16-19, 20-24), sexual orientation (men who have sex with men or men and women, men who have sex only with women, women who have sex with men or men and women, women who only have sex with women), ethnic group (Caucasian, Black, other) and age at which left education (16 or under, 17 or over). The subgroups will be analysed by inclusion of an interaction term between treatment group and the subgroup in the appropriate regression model for the outcome. Data will be presented by categories of subgroup using effect estimates and 99% confidence intervals. For each subgroup analysis, missing outcomes will be imputed consistent with the hypothesis of an interaction (33) .

Process outcomes

For peer review only - http://bmjopen.bmj.com/site/about/guidelines.xhtml

Candidate questionnaire items were identified for the measurement of the following constructs: attitudes towards partner notification; self–efficacy in telling a partner about an infection; self–efficacy in negotiating condom use; correct condom use self-efficacy; knowledge related to STIs. At four weeks, each of the five process outcomes were measured using the candidate questionnaire items (3 to 5 items per process outcome). Based on these items, we will carry out confirmatory factor analysis (CFA) to refine and identify a valid measurement model. For the main trial analyses, the final measurement model will be combined with the intervention group allocation, along with key baseline predictors, extending the measurement model to a Multiple Causes Multiple Indicators (MIMIC) model. This model will be used to estimate the effects of the intervention on the five hypothesised process outcomes.

Economic evaluation

The economic modelling required to assess the cost-effectiveness of the intervention will estimate the annual probability of members of the target group acquiring Chlamydia, gonorrhoea and NSU with and without the intervention (based on the experience of those in the control and intervention arms of the trial). Detailed information will be collected on the costs of delivering the intervention. Secondary sources will be used to estimate the future NHS costs avoided as a consequence of avoided infections.

Patient involvement

Patients were involved in the development of the intervention (15) and the design of the trial materials and follow-up procedures (34). A patient of also a member of the Trial Steering Committee. Young people will be involved in the dissemination of the trial results.

Monitoring

The safetxt trial will not have a separate Data Monitoring Committee (DMC). No interim analyses of intervention effects will be conducted since the trial is of a behavioural support intervention unlikely to cause harm, and therefore, there are no stopping rules. Analysis of intervention effects will be conducted once at the end of the trial. The sponsor may audit the trial per their own risk assessment and schedule.

In addition to collecting data on two adverse outcomes (involvement in car accident and experience of partner violence), participants will be asked an open-ended question in the 12 month questionnaire: “Did anything good or bad happen as a result of being involved in the study or receiving the text messages? Please describe.” The Clinical Trials Unit will keep detailed records of all adverse events reported. Reports will be reviewed by the Principal Investigator and

For peer review only - http://bmjopen.bmj.com/site/about/guidelines.xhtml

the TSC to consider intensity, causality and expectedness. As appropriate, these will be reported to the sponsor and the Ethics Committee.

Ethics and Dissemination

Ethical approval for this trial was provided by the NHS Health Research Authority - London – Riverside Research Ethics Committee (REC reference: 15/LO/1665) and the LSHTM. All trial participants will provide written informed consent prior to enrolment in the trial. Participants' can withdraw from the trial on request. Participant will receive usual NHS care there is no ancillary care.

Any modifications to the protocol will be approved by the ethics committee prior to implementation. Records of any important modifications will be submitted as an addendum the journal in which this protocol is published.

Trial results will be published open-access in peer-review journals. Authorship will be on the basis of meeting the criteria recommended by the International Committee of Medical Journal Editors (ICMJE).

After the publication of our main trial findings, the anonymised trial dataset will be available on request from the corresponding author.

DISCUSSION

STIs such as Chlamydia and gonorrhoea confer a heavy burden of disease among young people with long term sequelae such as infertility. Safer sex behaviours such as condom use, notifying partner(s) about an existing STI and STI testing reduce the risk of STI, but young people may lack the knowledge, confidence and skills needed to adopt these behaviours. Safer sex interventions delivered by mobile phone are acceptable to young people and show promise in increasing safer sex behaviours, but to date their effects on STI are not reliably known.

If it proves to be effective, this low-cost intervention could have an important impact in improving sexual health and reducing inequalities among young people in the UK. There is likely to be international interest in the impact of the intervention as short written messages delivered via mobile phones are increasingly used for behavioural support worldwide and sexually transmitted infections remain an important cause of morbidity and mortality.

For peer review only - http://bmjopen.bmj.com/site/about/guidelines.xhtml

Supplementary files

Supplementary File 1. Patient Information sheet

Supplementary File 2. Patient consent form

List of abbreviations

CTU – Clinical Trials Unit

GCP – Good Clinical Practice STI - Sexually transmitted infection

HIV – Human Immunodeficiency Virus

LSHTM - London School of Hygiene & Tropical Medicine

MICE – multiple imputation by chained equations

MSM – men who have sex with men

NAAT - nucleic acid amplification test

NSU – Non-specific urethritis

REFERENCES

1. Sonnenberg P, Clifton S, Beddows S, Field N, Soldan K, Tanton C, et al. Prevalence, risk factors, and uptake of interventions for sexually transmitted infections in Britain: findings from the National Surveys of Sexual Attitudes and Lifestyles (Natsal). Lancet. 2013;382(9907):1795-806.
2. Cates W, Jr., Wasserheit JN. Genital chlamydial infections: epidemiology and reproductive sequelae. Am J Obstet Gynecol. 1991;164(6 Pt 2):1771-81.
3. Hosenfeld CB, Workowski KA, Berman S, Zaidi A, Dyson J, Mosure D, et al. Repeat infection with Chlamydia and gonorrhea among females: a systematic review of the literature. Sex Transm Dis. 2009;36(8):478-89.
4. Sheringham J, Mann S, Simms I, Stafford M, Hart GJ, Raine R. It matters what you measure: a systematic literature review examining whether young people in poorer socioeconomic circumstances are more at risk of chlamydia. Sexually Transmitted Infections. 2013;89(2):175-80.
5. De Waroux OLP, Harris R, Hughes G, Crook P. The epidemiology of gonorrhoea in London: a Bayesian spatial modelling approach. Epidemiology and infection. 2014;142(01):211-20.
6. Hughes G, Nichols T, Peters L, Bell G, Leong G, Kinghorn G. Repeat infection with gonorrhoea in Sheffield, UK: predictable and preventable? Sexually Transmitted Infections. 2012.
7. Free C, Roberts IG, Abramsky T, Fitzgerald M, Wensley F. A systematic review of randomised controlled trials of interventions promoting effective condom use. J Epidemiol Community Health. 2011;65(2):100-10.
8. French RS, Bonell C, Wellings K, Weatherburn P. An exploratory review of HIV prevention mass media campaigns targeting men who have sex with men. BMC Public Health. 2014;14:616.
9. Estcourt C SL, Mercer CH, Copas A, Muniia P, Rait G, et al. Can accelerated partner therapy (APT) improve outcomes of partner notification for women diagnosed with genital chlamydia in

For peer review only - http://bmjopen.bmj.com/site/about/guidelines.xhtml

primary care settings: A pilot randomized controlled trial in general practice and community sexual health services. Sexually Transmitted Diseases Conference; Conference start: 20140609 Conference end: 20140612; Atlanta, GA United States2014. p. S32-S3.
10. Control ECfDPa. Public health benefits of partner notification for sexually transmitted infections and HIV. Stockholm: European Centre for Disease Prevention and Control: 2013.
11. Guerriero C, Cairns J, Roberts I, Rodgers A, Whittaker R, Free C. The cost-effectiveness of smoking cessation support delivered by mobile phone text messaging: Txt2stop. Eur J Health Econ. 2013;14(5):789-97.
12. Free C, Knight R, Robertson S, Whittaker R, Edwards P, Zhou W, et al. Smoking cessation support delivered via mobile phone text messaging (txt2stop): a single-blind, randomised trial. Lancet. 2011;378(9785):49-55.
13. Free C, Phillips G, Galli L, Watson L, Felix L, Edwards P, et al. The effectiveness of mobile-health technology-based health behaviour change or disease management interventions for health care consumers: a systematic review. PLoS Med. 2013;10(1):e1001362.
14. Burns K, Keating P, Free C. A Systematic Review of mHealth RCTs for Sexual Health. BMC Public Health. 2016;16:778.
15. Free C, McCarthy O, French R, Wellings K, Michie S, Roberts I, et al. Can text messages increase safer sex behaviours in young people? Intervention development and pilot randomised controlled trial. Health Technol Assess 2016;20(57).
16. de Tolly K, Skinner D, Nembaware V, Benjamin P. Investigation into the use of short message services to expand uptake of human immunodeficiency virus testing, and whether content and dosage have impact. Telemed J E Health. 2012;18(1):18-23.
17. Gold J, Lim MS, Hellard ME, Hocking JS, Keogh L. What's in a message? Delivering sexual health promotion to young people in Australia via text messaging. BMC public health. 2010;10:792.
18. Lim MS, Hocking JS, Aitken CK, Fairley CK, Jordan L, Lewis JA, et al. Impact of text and email messaging on the sexual health of young people: a randomised controlled trial. J Epidemiol Community Health. 2012;66(1):69-74.
19. Field N, Mercer CH, Sonnenberg P, Tanton C, Clifton S, Mitchell KR, et al. Associations between health and sexual lifestyles in Britain: findings from the third National Survey of Sexual Attitudes and Lifestyles (Natsal-3). Lancet. 2013;382(9907):1830-44.
20. Downing SG, Cashman C, McNamee H, Penney D, Russell DB, Hellard ME. Increasing chlamydia test of re-infection rates using SMS reminders and incentives. Sex Transm Infect. 2013;89(1):16-9.
21. Mugo PM, Wahome EW, Gichuru EN, Mwashigadi GM, Thiong'o AN, Prins HA, et al. Effect of Text Message, Phone Call, and In-Person Appointment Reminders on Uptake of Repeat HIV Testing among Outpatients Screened for Acute HIV Infection in Kenya: A Randomized Controlled Trial. PLoS ONE [Electronic Resource]. 2016;11:e0153612.
22. Odeny TA, Bailey RC, Bukusi EA, Simoni JM, Tapia KA, Yuhas K, et al. Effect of text messaging to deter early resumption of sexual activity after male circumcision for HIV prevention: a randomized controlled trial. J Acquir Immune Defic Syndr. 2014;65(2):e50-7.
23. Suffoletto B, Akers A, McGinnis KA, Calabria J, Wiesenfeld HC, Clark DB. A sex risk reduction text-message program for young adult females discharged from the emergency department. J Adolesc Health. 2013;53(3):387-93.
24. Jones R, Hoover DR, Lacroix LJ. A randomized controlled trial of soap opera videos streamed to smartphones to reduce risk of sexually transmitted human immunodeficiency virus (HIV) in young urban African American women. Nurs Outlook. 2013;61(4):205-15 e3.
25. Thomas N, Murray E, Rogstad KE. Confidentiality is essential if young people are to access sexual health services. Int J STD AIDS. 2006;17(8):525-9.
26. French RS, McCarthy O, Baraitser P, Wellings K, Bailey JV, Free C. Young People's Views and Experiences of a Mobile Phone Texting Intervention to Promote Safer Sex Behavior. JMIR mHealth and uHealth. 2016;4(2).

For peer review only - http://bmjopen.bmj.com/site/about/guidelines.xhtml

27. McCarthy OL, French RS, Baraitser P, Roberts I, Rathod SD, Devries K, et al. Safetxt: a pilot randomised controlled trial of an intervention delivered by mobile phone to increase safer sex behaviours in young people. BMJ Open. 2016;6(12).
28. Michie S, M van Stralen M, West R. The behaviour change wheel: A new method for characterising and designing behaviour change interventions

2011;6:42.
29. Michie S, Wood C, Johnston M, Abraham C, Francis J, Hardeman W. Behaviour change techniques: the development and evaluation of a taxonomic method for reporting and describing behaviour change interventions. Health Technology Assessment. 2015;19(99):1-188.
30. Data Protection Act. 1998.
31. Sterne JA, White IR, Carlin JB, Spratt M, Royston P, Kenward MG, et al. Multiple imputation for missing data in epidemiological and clinical research: potential and pitfalls. BMJ. 2009;338:b2393.
32. Cro S, Carpenter J, Kenward MIasataa. Information anchored sensitivity analysis: theory and application. Journal of the Royal Statistical Society. 2018;182:623-45.
33. Carpenter JR, Kenward M. Multiple imputation and its application. Chichester: Wiley; 2013.
34. McCarthy O, French R, Roberts I, Free C. Simple steps to develop trial follow-up procedures. Trials. 2016;17(28).

Figures

Figure 1. Trial flow chart

Figure 2. Schedule of enrolment, interventions and assessments

Authors' contributions

CF conceived the study. CF, OM, RF, FH, KW KD, PB, SM and JB worked on the development of the intervention. CF took overall responsibility for the design and conduct of the trial and writing the protocol. All authors contributed to the trial design. PE, TC, JC and GP provided statistical expertise for the trial analysis or the Generalised Latent Variable Modelling framework. RK and KP are responsible for the day-to-day management of the trial. All authors contributed to the refinement of the study protocol and approved the manuscript

Funding

This trial is funded by the National Institute for Health Research Public Health Research (NIHR PHR) Programme (project number 14/182/07). The funding body have no role in any aspect of the design and conduct of this study, or the decision to submit the report for publication.

Competing interests

The authors declare that they have no competing interests.

Ethics approval

For peer review only - http://bmjopen.bmj.com/site/about/guidelines.xhtml

Ethics approval for this trial was provided by the NHS Health Research Authority - London – Riverside Research Ethics Committee (REC reference: 15/LO/1665) and the LSHTM.

Trial Sponsor

London School of Hygiene & Tropical Medicine is the main research sponsor for this trial. The sponsor had no role in any aspect of the design and conduct of this study, or the decision to submit the report for publication. For further information regarding the sponsorship conditions, please contact the Research Governance and Integrity Office:

London School of Hygiene & Tropical Medicine, Keppel Street, London, WC1E 7HT

Tel: +44 207 927 2626 Email: patricia.henley@lshtm.ac.uk

Trial Steering Committee

The Trial Steering Committee (TSC) may recommend changes to the overall trial management processes for which there is evidence the current process may compromise the conduct of the trial or the safety of participants. The members of the TSC are: Professor Pippa Oakeshott (Chair), Dr Andrew Copas, Professor Michael Ussher, Mr Colum McGrady, Dr Michael Brady, and Professor Caroline Free.

Word count 3997

For peer review only

For peer review only - http://bmjopen.bmj.com/site/about/guidelines.xhtml

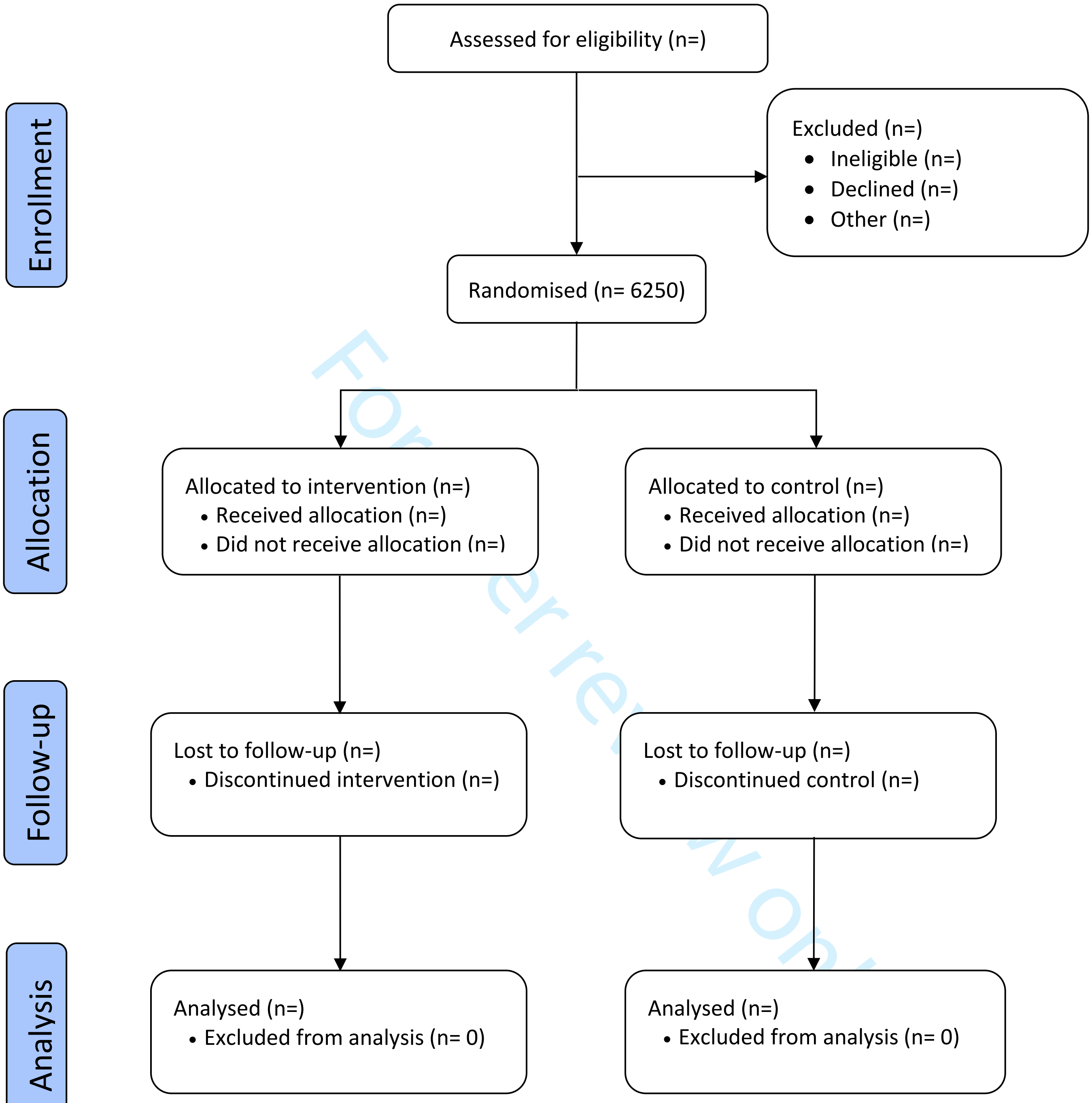
Enrollment
Assessed for eligibility (n=)
Excluded (n=)
• Ineligible (n=)
• Declined (n=)
• Other (n=)
Randomised (n= 6250)
Allocation
Allocated to intervention (n=)
• Received allocation (n=)
• Did not receive allocation (n=)
Allocated to control (n=)
• Received allocation (n=)
• Did not receive allocation (n=)
Follow-up
Lost to follow-up (n=)
• Discontinued intervention (n=)
Lost to follow-up (n=)
• Discontinued control (n=)
Analysis
Analysed (n=)
• Excluded from analysis (n= 0)
Analysed (n=)
• Excluded from analysis (n= 0)

Figure 2. Schedule of enrolment, interventions, and assessments.

	STUDY PERIOD				
	**Enrolment**	**Allocation**	**Post-allocation**		**Close-out**
**TIMEPOINT****	**0**	**0**	***Wk 4***	***Wk 52***	***Wk 76***
**ENROLMENT:**					
**Eligibility screen**	X				
**Informed consent**	X				
**Allocation**		X			
**INTERVENTIONS:**					
safetxt text messages					
control text messages					
**ASSESSMENTS:**					
Socio-demographic characteristics	X				
Recent sexual behaviour	X		X	X	X
STI diagnosis	X				
Correct treatment of STI			X		
Partner notification			X		
Attitudes, self-efficacy, knowledge			X		
STI test (postal NAAT)				X	X
STI testing prior to sex with new partner				X	X
Number of text messages read				X	X
Anyone else read text messages				X	X
Experience of car accident				X	X
Experience of partner violence				X	X

*

For peer review only

A randomised controlled trial of a safer sex intervention delivered through mobile phone messaging

Local site logo

Participant's Information Sheet
safetxt: a randomised controlled trial of a safer sex intervention

We are inviting you to take part in a research study. Before you decide, it is important that you know why we are doing the study and what is involved. Please read the following information carefully.

What is the study?
This study is testing whether text messages providing information and tips to increase safer sex helps young people adopt safer sex behaviours.

Why have I been chosen?
You are aged 16-24 and have recently received a positive test result for Chlamydia, gonorrhoea or had a non specific urethritis diagnosis.

Do I have to take part?
No, it is up to you to decide whether to take part.

What will happen if I take part?
After you have had all your questions answered and have agreed to take part, we will ask you to complete a consent form. You can do this on our secure and confidential study website, by filling out a paper version, by text message or by email. We will then ask you to complete a short confidential questionnaire providing details about yourself.

After you have completed the questionnaire, **an automated computer system will put you into one of two groups by chance (randomly)**. One group will receive safer sex text messages for 1-2 weeks, then about 5 messages a month for 11 months. The other group will receive text messages asking you to confirm your contact details. These messages will not be about sexual health. This group will receive one message a month for 12 months.

We will ask you to complete the questionnaire again at 1 and 12 months after signing up. This is to see how things have been for you after joining the study. Also at 12 months we will ask you to provide a sample to test for Chlamydia and gonorrhoea. The testing kit will be sent to you by post. **Participating in the study involves returning one urine sample or self-taken swab to test for Chlamydia and gonorrhoea.**

Will you compensate me for the time this takes?
We will offer you £5 as compensation for your time at each follow up: for the 1 month questionnaire and the 12 month questionnaire. You will be compensated for your time for returning your 12 month sample.

We do not charge for text messages we send to you.

What are the alternatives?
You do not have to take part.

What are the possible disadvantages in taking part?
Completing the questionnaires and providing a sample will take up some of your time. It is possible that the messages we send could be read by someone else. If you are concerned about this, you can password-lock your phone and delete the messages after you read them.

For peer review only - http://bmjopen.bmj.com/site/about/guidelines.xhtml

What are the possible benefits of taking part?

We cannot promise that your health will benefit from taking part but you may find the messages helpful and you may learn about safer sex behaviours.

What happens when this study stops?

When the study stops, we will look at the data collected to see if the text messages helped increase safer sex behaviours.

What will happen if I don't want to carry on with the study?

You can stop receiving text messages by texting STOP or pause the text messages by texting PAUSE. You can withdraw at any time by letting the Clinical Trials Unit know (safetxt@LSHTM.ac.uk or freephone 0800 008 7197). You do not have to give a reason for wanting to withdraw.

What if there is a problem?

You can contact the Clinical Trials Unit if there is a problem. If you would like to make a formal complaint, contact Dr Caroline Free who will follow the complaints procedure.

Will my taking part in this study be kept confidential?

Yes. Your answers to the questionnaires will be stored anonymously and your contact details and Chlamydia and gonorrhoea test will be kept confidential. You will be allocated a unique study number when you enrol in the study. We will not inform your parents about your involvement in this research. If you were tested for sexually transmitted infections during the study, we will ask your clinic or GP for your test results. We will also ask for information on whether or not a partner was treated. To keep the messages confidential, password-lock your phone and delete messages after you read them.

Your records will be made available to people authorised to work on the study. They may also need to be made available to people for ensuring that the study is carried our correctly.

With your permission, we will ask your clinic or GP for your test results for sexually transmitted infections after you have finished participating in the study. This is to allow the study researchers to look into long term effects about your sexual health.

What will happen to the samples that I give?

You will post your sample directly to a laboratory to test for Chlamydia and gonorrhoea infection. The laboratory will destroy the samples after they have finished testing. You will be notified if the test shows that there is Chlamydia or gonorrhoea infection. The clinic where you signed up to the study will contact you to arrange treatment.

How long will your data be stored for?

Your data will be kept for 10 years after the trial is completed. However, in accordance with the Data Protection Act of 1998, any personal identifiable data will not be kept longer than necessary and will be deleted within 3 months after you have finished the study.

What will happen to the results of the research study?

The results will be published in a scientific journal so that other people know about it. If you would like a copy of the results please contact the Clinical Trials Unit.

If you want to receive the safer sex messages after your participation in the study, please let us know and we will send them to you.

If the results of the study show that the messages have helped, they will be made available to all young people.

Who is organising and funding the research?

The study is run by Dr Caroline Free (Chief Investigator) from the London School of Hygiene and Tropical Medicine, at the University of London. The UK National Institute for Health Research is funding the trial.

For peer review only - http://bmjopen.bmj.com/site/about/guidelines.xhtml

Who has reviewed the trial?

London Riverside NHS Medical Research Ethics Committee.

Thank you for taking the time to consider taking part.

If you would like further information please contact the local Research Nurse or Clinical Trials Unit (details below)

The team contact details:

INSERT LOCAL CONTACT DETAILS

If you have any questions about the trial, please email: safetxt@LSHTM.ac.uk or Freephone: 0800 008 7197

only

For peer review only - http://bmjopen.bmj.com/site/about/guidelines.xhtml

safetxt — A randomised controlled trial of a safer sex intervention delivered through mobile phone messaging

Hospital logo to be inserted

Consent Form

Chief Investigator: Dr Caroline Free

Name of local researcher: *(Please insert local details)*

Please **initial box**

I confirm that I have read and understand the information sheet [version 8, 16th April 2018] for the above study. I have had the opportunity to consider the information, ask questions and have had these answered satisfactorily. ☐

I understand that participation in this study is my choice and that I am free to withdraw at any time without giving any reason, without my medical care or legal rights being affected. ☐

I understand that I should not read or send text messages while driving a vehicle. ☐

I understand that relevant sections of data collected during the study may be looked at by the NHS Trust or LSHTM, where it is relevant to my taking part in this research. I give permission for these individuals to have access to my records. ☐

I agree to the study researchers letting my clinic know of any positive test results I receive so they can arrange treatment. ☐

I agree that my clinic/GP can provide the researchers with my test results for sexually transmitted infections. ☐

If I give my partner(s) details to my clinic, I agree to the researchers being told if they have been tested. ☐

I understand that all information I provide will remain confidential in accordance with the Data Protection Act of 1998. ☐

I understand that the information collected about me will be used to support other research in the future, and may be shared anonymously with other researchers. ☐

I agree to take part in the above study. ☐

<u>Optional</u>

After the study, I agree that my clinic/GP can provide the researchers with my sexually transmitted infection results. ☐

Name of Patient	Date <u>and</u> time	Signature
Name of researcher taking consent	Date <u>and</u> time	Signature

For peer review only - http://bmjopen.bmj.com/site/about/guidelines.xhtml

SPIRIT
STANDARD PROTOCOL ITEMS: RECOMMENDATIONS FOR INTERVENTIONAL TRIALS

SPIRIT 2013 Checklist: Recommended items to address in a clinical trial protocol and related documents*

Section/item	Item No	Description	Addressed on page number
**Administrative information**			
Title	1	Descriptive title identifying the study design, population, interventions, and, if applicable, trial acronym	1
Trial registration	2a	Trial identifier and registry name. If not yet registered, name of intended registry	3
	2b	All items from the World Health Organization Trial Registration Data Set	3
Protocol version	3	Date and version identifier	3
Funding	4	Sources and types of financial, material, and other support	18
Roles and responsibilities	5a	Names, affiliations, and roles of protocol contributors	18
	5b	Name and contact information for the trial sponsor	19
	5c	Role of study sponsor and funders, if any, in study design; collection, management, analysis, and interpretation of data; writing of the report; and the decision to submit the report for publication, including whether they will have ultimate authority over any of these activities	19
	5d	Composition, roles, and responsibilities of the coordinating centre, steering committee, endpoint adjudication committee, data management team, and other individuals or groups overseeing the trial, if applicable (see Item 21a for data monitoring committee)	19

For peer review only

Introduction

Background and rationale	6a	Description of research question and justification for undertaking the trial, including summary of relevant studies (published and unpublished) examining benefits and harms for each intervention	5-6
	6b	Explanation for choice of comparators	8-9
Objectives	7	Specific objectives or hypotheses	6
Trial design	8	Description of trial design including type of trial (eg, parallel group, crossover, factorial, single group), allocation ratio, and framework (eg, superiority, equivalence, noninferiority, exploratory)	6

Methods: Participants, interventions, and outcomes

Study setting	9	Description of study settings (eg, community clinic, academic hospital) and list of countries where data will be collected. Reference to where list of study sites can be obtained	6
Eligibility criteria	10	Inclusion and exclusion criteria for participants. If applicable, eligibility criteria for study centres and individuals who will perform the interventions (eg, surgeons, psychotherapists)	6-7
Interventions	11a	Interventions for each group with sufficient detail to allow replication, including how and when they will be administered	8-9
	11b	Criteria for discontinuing or modifying allocated interventions for a given trial participant (eg, drug dose change in response to harms, participant request, or improving/worsening disease)	14
	11c	Strategies to improve adherence to intervention protocols, and any procedures for monitoring adherence (eg, drug tablet return, laboratory tests)	n/a
	11d	Relevant concomitant care and interventions that are permitted or prohibited during the trial	9
Outcomes	12	Primary, secondary, and other outcomes, including the specific measurement variable (eg, systolic blood pressure), analysis metric (eg, change from baseline, final value, time to event), method of aggregation (eg, median, proportion), and time point for each outcome. Explanation of the clinical relevance of chosen efficacy and harm outcomes is strongly recommended	9-10
Participant timeline	13	Time schedule of enrolment, interventions (including any run-ins and washouts), assessments, and visits for participants. A schematic diagram is highly recommended (see Figure)	Figure 2

For peer review only - http://bmjopen.bmj.com/site/about/guidelines.xhtml

Sample size	14	Estimated number of participants needed to achieve study objectives and how it was determined, including clinical and statistical assumptions supporting any sample size calculations	12
Recruitment	15	Strategies for achieving adequate participant enrolment to reach target sample size	7

Methods: Assignment of interventions (for controlled trials)

Allocation:

Sequence generation	16a	Method of generating the allocation sequence (eg, computer-generated random numbers), and list of any factors for stratification. To reduce predictability of a random sequence, details of any planned restriction (eg, blocking) should be provided in a separate document that is unavailable to those who enrol participants or assign interventions	7
Allocation concealment mechanism	16b	Mechanism of implementing the allocation sequence (eg, central telephone; sequentially numbered, opaque, sealed envelopes), describing any steps to conceal the sequence until interventions are assigned	7
Implementation	16c	Who will generate the allocation sequence, who will enrol participants, and who will assign participants to interventions	7
Blinding (masking)	17a	Who will be blinded after assignment to interventions (eg, trial participants, care providers, outcome assessors, data analysts), and how	7
	17b	If blinded, circumstances under which unblinding is permissible, and procedure for revealing a participant's allocated intervention during the trial	n/a

Methods: Data collection, management, and analysis

Data collection methods	18a	Plans for assessment and collection of outcome, baseline, and other trial data, including any related processes to promote data quality (eg, duplicate measurements, training of assessors) and a description of study instruments (eg, questionnaires, laboratory tests) along with their reliability and validity, if known. Reference to where data collection forms can be found, if not in the protocol	11
	18b	Plans to promote participant retention and complete follow-up, including list of any outcome data to be collected for participants who discontinue or deviate from intervention protocols	11

Data management	19	Plans for data entry, coding, security, and storage, including any related processes to promote data quality (eg, double data entry; range checks for data values). Reference to where details of data management procedures can be found, if not in the protocol	11-12
Statistical methods	20a	Statistical methods for analysing primary and secondary outcomes. Reference to where other details of the statistical analysis plan can be found, if not in the protocol	12-14
	20b	Methods for any additional analyses (eg, subgroup and adjusted analyses)	13
	20c	Definition of analysis population relating to protocol non-adherence (eg, as randomised analysis), and any statistical methods to handle missing data (eg, multiple imputation)	13
**Methods: Monitoring**			
Data monitoring	21a	Composition of data monitoring committee (DMC); summary of its role and reporting structure; statement of whether it is independent from the sponsor and competing interests; and reference to where further details about its charter can be found, if not in the protocol. Alternatively, an explanation of why a DMC is not needed	14
	21b	Description of any interim analyses and stopping guidelines, including who will have access to these interim results and make the final decision to terminate the trial	14
Harms	22	Plans for collecting, assessing, reporting, and managing solicited and spontaneously reported adverse events and other unintended effects of trial interventions or trial conduct	14
Auditing	23	Frequency and procedures for auditing trial conduct, if any, and whether the process will be independent from investigators and the sponsor	14
**Ethics and dissemination**			
Research ethics approval	24	Plans for seeking research ethics committee/institutional review board (REC/IRB) approval	15
Protocol amendments	25	Plans for communicating important protocol modifications (eg, changes to eligibility criteria, outcomes, analyses) to relevant parties (eg, investigators, REC/IRBs, trial participants, trial registries, journals, regulators)	15

Consent or assent	26a	Who will obtain informed consent or assent from potential trial participants or authorised surrogates, and how (see Item 32)	7
	26b	Additional consent provisions for collection and use of participant data and biological specimens in ancillary studies, if applicable	n/a
Confidentiality	27	How personal information about potential and enrolled participants will be collected, shared, and maintained in order to protect confidentiality before, during, and after the trial	11-12
Declaration of interests	28	Financial and other competing interests for principal investigators for the overall trial and each study site	18
Access to data	29	Statement of who will have access to the final trial dataset, and disclosure of contractual agreements that limit such access for investigators	15
Ancillary and post-trial care	30	Provisions, if any, for ancillary and post-trial care, and for compensation to those who suffer harm from trial participation	n/a
Dissemination policy	31a	Plans for investigators and sponsor to communicate trial results to participants, healthcare professionals, the public, and other relevant groups (eg, via publication, reporting in results databases, or other data sharing arrangements), including any publication restrictions	15
	31b	Authorship eligibility guidelines and any intended use of professional writers	15
	31c	Plans, if any, for granting public access to the full protocol, participant-level dataset, and statistical code	n/a
**Appendices**			
Informed consent materials	32	Model consent form and other related documentation given to participants and authorised surrogates	Supplementary Files 1&2
Biological specimens	33	Plans for collection, laboratory evaluation, and storage of biological specimens for genetic or molecular analysis in the current trial and for future use in ancillary studies, if applicable	11

*It is strongly recommended that this checklist be read in conjunction with the SPIRIT 2013 Explanation & Elaboration for important clarification on the items. Amendments to the protocol should be tracked and dated. The SPIRIT checklist is copyrighted by the SPIRIT Group under the Creative Commons "Attribution-NonCommercial-NoDerivs 3.0 Unported" license.

For peer review only - http://bmjopen.bmj.com/site/about/guidelines.xhtml

BMJ Open

safetxt: a safer sex intervention delivered by mobile phone messaging on sexually transmitted infections (STI) among young people in the UK- protocol for a randomised controlled trial

Journal:	*BMJ Open*
Manuscript ID	bmjopen-2019-031635.R1
Article Type:	Protocol
Date Submitted by the Author:	05-Nov-2019

Complete List of Authors:	Free, Caroline; London School of Hygiene and Tropical Medicine, Population Health<br>McCarthy, Ona; London School of Hygiene and Tropical Medicine, Population Health<br>Palmer, Melissa; London School of Hygiene and Tropical Medicine, Population Health<br>Knight, Rosemary; London School of Hygiene and Tropical Medicine, Clinical Trials Unit, MSD<br>Edwards, Phil; London School of Hygiene and Tropical Medicine, Population Health<br>French, Rebecca ; London School of Hygiene and Tropical Medicine, Social and Environmental Health Research<br>Baraitser, Paula; King's College London, Centre for Global Health<br>Hickson, Ford; London School of Hygiene and Tropical Medicine, Sigma Research<br>Wellings, Kaye; London School of Hygiene and Tropical Medicine, Social and Environmental Health Research<br>Roberts, Ian; London School of Hygiene and Tropical Medicine, Population Health<br>Bailey, Julia; University College London, Primary Care and Population Health<br>Hart, Graham; University College London, Department of Infection and Population Health<br>Michie, Susan; University College London, Centre for Outcomes Research and Effectivenes<br>Clayton, Tim; London School of Hygiene and Tropical Medicine<br>Ploubidis, George; University College London Institute of Education<br>Carpenter, James; London School of Hygiene and Tropical Medicine<br>Turner, Katy; University of Bristol<br>Devries, Karen; London School of Hygiene and Tropical Medicine<br>Potter, Kimberley; London School of Hygiene and Tropical Medicine

**Primary Subject Heading**:	Sexual health
Secondary Subject Heading:	Communication, General practice / Family practice
Keywords:	sexually transmitted infection, sexual behaviour, text message, mHealth,

	randomised controlled trial, young people

SCHOLARONE™
Manuscripts

I, the Submitting Author has the right to grant and does grant on behalf of all authors of the Work (as defined in the below author licence), an exclusive licence and/or a non-exclusive licence for contributions from authors who are: i) UK Crown employees; ii) where BMJ has agreed a CC-BY licence shall apply, and/or iii) in accordance with the terms applicable for US Federal Government officers or employees acting as part of their official duties; on a worldwide, perpetual, irrevocable, royalty-free basis to BMJ Publishing Group Ltd ("BMJ") its licensees and where the relevant Journal is co-owned by BMJ to the co-owners of the Journal, to publish the Work in this journal and any other BMJ products and to exploit all rights, as set out in our licence.

The Submitting Author accepts and understands that any supply made under these terms is made by BMJ to the Submitting Author unless you are acting as an employee on behalf of your employer or a postgraduate student of an affiliated institution which is paying any applicable article publishing charge ("APC") for Open Access articles. Where the Submitting Author wishes to make the Work available on an Open Access basis (and intends to pay the relevant APC), the terms of reuse of such Open Access shall be governed by a Creative Commons licence – details of these licences and which Creative Commons licence will apply to this Work are set out in our licence referred to above.

Other than as permitted in any relevant BMJ Author's Self Archiving Policies, I confirm this Work has not been accepted for publication elsewhere, is not being considered for publication elsewhere and does not duplicate material already published. I confirm all authors consent to publication of this Work and authorise the granting of this licence.

For peer review only

Title Page

safetxt: a safer sex intervention delivered by mobile phone messaging on sexually transmitted infections (STI) among young people in the UK- protocol for a randomised controlled trial.

Authors

Caroline Free, Department of Population Health, Faculty of Epidemiology and Population Health, London School of Hygiene & Tropical Medicine, Keppel Street, London, WC1E 7HT. caroline.free@lshtm.ac.uk

Ona L McCarthy, Department of Population Health, Faculty of Epidemiology and Population Health, London School of Hygiene & Tropical Medicine, Keppel Street, London, WC1E 7HT. ona.mccarthy@lshtm.ac.uk

Melissa Palmer, Department of Population Health, Faculty of Epidemiology and Population Health, London School of Hygiene & Tropical Medicine, Keppel Street, London, WC1E 7HT. melissa.palmer@lshtm.ac.uk

Rosemary Knight, Clinical Trials Unit. Department of Medical Statistics, Faculty of Epidemiology and Population Health, London School of Hygiene & Tropical Medicine, Keppel Street, London, WC1E 7HT. rosemary.knight@lshtm.ac.uk

Phil Edwards, Department of Population Health, Faculty of Epidemiology and Population Health, London School of Hygiene & Tropical Medicine, Keppel Street, London, WC1E 7HT. phil.edwards@lshtm.ac.uk

Rebecca French, Department of Public Health Environments and Society, Faculty of Public Health and Policy, London School of Hygiene & Tropical Medicine, 15-17 Tavistock Place, London, WC1H 9SH. rebecca.french@lshtm.ac.uk

Paul Baraitser, King's Centre for Global Health, King's College London, Camberwell Sexual Health Centre, 100 Denmark Hill, London, SE5 9RS. paula_baraitser@mac.com

Ford Hickson, Department of Social and Environmental Health Research, Faculty of Public Health and Policy, London School of Hygiene & Tropical Medicine, 15-17 Tavistock Place, London, WC1H 9SH. ford.hickson@lshtm.ac.uk

Kaye Wellings, Department of Public health Environments and Society, Faculty of Public Health and Policy, London School of Hygiene & Tropical Medicine, 15-17 Tavistock Place, London, WC1H 9SH. kaye.wellings@lshtm.ac.uk

For peer review only - http://bmjopen.bmj.com/site/about/guidelines.xhtml

For peer review only

Ian Roberts, Department of Population Health, Faculty of Epidemiology and Population Health, London School of Hygiene & Tropical Medicine, Keppel Street, London, WC1E 7HT. ian.roberts@ucl.ac.uk

Julia V Bailey, Department of Primary Care and Population Health, University College London, Gower Street, London, WC1E 6BT. julia.bailey@ucl.ac.uk

Graham Hart, Faculty of Population Health Sciences, University College London, Gower Street, London, WC1E 6BT. g.hart@ucl.ac.uk

Susan Michie, Department of Clinical, Educational and Health Psychology, University College London, Gower Street, London, WC1E 6BT. s.michie@ucl.ac.uk

Tim Clayton. Department of Medical Statistics. Faculty of Epidemiology and Population Health, London School of Hygiene & Tropical Medicine, Keppel Street, London, WC1E 7HT. tim.clayton@lshtm.ac.uk

George Ploubidis, Department of Social Science, Institute of Education, University College London, Gower Street, London, WC1E 6BT. g.ploubidis@ucl.ac.uk

James Carpenter, Department of Medical Statistics. Faculty of Epidemiology and Population Health, London School of Hygiene & Tropical Medicine, Keppel Street, London, WC1E 7HT. james.carpenter@lshtm.ac.uk

Katy Turner, Bristol Veterinary School, University of Bristol, Churchill Building, Langford Campus, Bristol, BS40 5DU katy.turner@bristol.ac.uk

Karen Devries, Faculty of Public Health and Policy. LSHTM, 15-17 Tavistock Place, London WC1H 9SH. karen.devries@lshtm.ac.uk

Kimberley Potter, Clinical Trials Unit. Department of Medical Statistics, Faculty of Epidemiology and Population Health, London School of Hygiene & Tropical Medicine, Keppel Street, London, WC1E 7HT. kimberley.potter@lshtm.ac.uk

For peer review only - http://bmjopen.bmj.com/site/about/guidelines.xhtml

Abstract

Introduction

Young people aged 16-24 have the highest prevalence of genital chlamydia and gonorrhoea compared to other age groups and re-infection rates following treatment are high. Long-term adverse health effects include sub-fertility and ectopic pregnancy, particularly among those with repeated infections. We developed the safetxt intervention delivered by text message to reduce sexually transmitted infection (STI) by increasing partner notification, condom use and (STI) testing among young people in the United Kingdom (UK).

Methods and analysis

A single blind randomised trial to reliably establish the effect of the safetxt intervention on chlamydia and gonorrhoea infection at one year. We will recruit 6250 people aged 16-24 years who have recently been diagnosed with chlamydia, gonorrhoea or non-specific urethritis from health services in the UK. Participants will be allocated to receive the safetxt intervention (text messages designed to promote safer sexual health behaviours) or to receive the control text messages (monthly messages asking participants about changes in contact details) by an automated remote online randomisation system.

The primary outcome will be the cumulative incidence of chlamydia and gonorrhoea infection at one year assessed by nucleic acid amplification tests. Secondary outcomes include partner notification, correct treatment of infection, condom use, and STI testing prior to sex with new partners.

Ethics and dissemination

Ethics approval was obtained from NHS Health Research Authority - London – Riverside Research Ethics Committee (REC reference: 15/LO/1665) and the LSHTM. We will submit the results of the trial for publication in peer reviewed journals.

Trial registration

International Standard Randomised Controlled Trials Number: ISRCTN64390461. Registered on 17th March 2016. *World Health Organization trial registration dataset*

Available at: http://apps.who.int/trialsearch/Trial2.aspx?TrialID=ISRCTN64390461

Trial Protocol version: 12 19th July 2018

Please note that you cannot begin randomisation until given the green light by the sponsor.

For peer review only - http://bmjopen.bmj.com/site/about/guidelines.xhtml

Strengths and limitations of this study

- The study design is a large randomised trial to assess the effect of 'safetxt', a safer sex intervention delivered by text message, on objectively measured STI (chlamydia and gonorrhoea) infection at one year.
- The use of an independent remote computer-based randomisation system linked to an automated message delivery system ensures allocation concealment.
- The secondary outcomes will assess the effect of the intervention on safer sex behaviours: partner notification, condom use and testing before sex with a new partner.
- The process evaluation will explore which intervention components are effective and has the potential to generate general principles to inform similar interventions in the future.
- The inability to blind participants receiving a behavioural intervention is a limitation of the trial, which could introduce bias in the ascertainment of self-reported outcomes.

Keywords

Sexually transmitted infection (STI); sexual behaviour; young people; mHealth; randomised controlled trial, text message.

For peer review only

INTRODUCTION

Younger people aged 16-24 bear the heaviest burden of sexually transmitted infections (STIs) such as chlamydia and gonorrhoea, and their long-term adverse health effects including ectopic pregnancy and subfertility (1, 2). The risk of adverse health effects increases with repeated infections, and re-infection rates following treatment are high: up to 30% for chlamydia and 12% for gonorrhoea at one year (3). Those with an STI are more likely to acquire further STIs and HIV, if exposed. There are marked inequalities in sexual health; STIs are positively associated with lower educational level (4) and living in more deprived areas (1, 5, 6).

Partner notification, condom use and STI testing can reduce infection and re-infection. There is some evidence that existing interventions delivered face-to-face that target condom use and/or STI testing may be effective, but they are limited in their reach or too costly for widespread application (7). Existing interventions delivered via the media have high reach but their effects have yet to be established (8). Effective ways to increase partner notification in specialist and primary care settings are needed (9, 10).

Mobile phones have the potential to provide effective, low cost health behaviour support (11, 12). However, evidence for the effect of mobile phone support for safer sex behaviours such as condom use, partner notification and STI testing is equivocal (13-15). Interventions have targeted testing for STI (16-21), delaying resumption of sexual activity until 42 days after circumcision (22) and condom use (17, 18, 23, 24), but none have aimed to increase partner notification. The effect of mobile phone based interventions on STI is not known.

In the UK, 99% of 16-24 year olds are mobile phone users (ONS, 2012) and mobile phone ownership is high across all socio-economic groups. In research leading to this trial, it was demonstrated that interactive support via text message is particularly acceptable in the area of sexual health intervention (15, 25, 26).

The safetxt trial builds on the successful intervention development work and pilot trial (15, 26, 27). To develop the intervention, we convened a working group including experts in health psychology, contraception research, contraception service provision, IT and the development of interventions delivered by mobile phone. The intervention messages were developed based on: behaviour change theory; evidence-based behaviour change techniques; the content of effective face-to-face safer sex interventions; the factors known to influence safer sex behaviours; the views of 82 young people collected in focus groups and a questionnaire completed by 100 people aged 16-24 (15). The theory and evidence-based intervention is designed to reduce STIs in young

For peer review only - http://bmjopen.bmj.com/site/about/guidelines.xhtml

people by supporting them in telling a partner about an infection, using condoms and obtaining testing before unprotected sex with a new partner. In a qualitative study with young people, recipients reported that the tone, language, content, and frequency of messages was appropriate (26). Messages reportedly increased knowledge and confidence in how to use condoms and reduced stigma enabling them to tell a partner about an STI. Sharing messages with their partner enabled participants to negotiate condom use.

The pilot trial demonstrated that methods of recruitment, randomisation, intervention delivery and follow-up were successful and that a full-scale randomised controlled trial of the safetxt intervention is feasible (27).

Objectives

The primary objective of this trial is to quantify the effects of the safetxt intervention compared to a control group receiving usual care and messages about trial participation on chlamydia or gonorrhoea infection at one year. Secondary objectives are to determine the effects of safetxt on partner notification and condom use at 4 weeks and on condom use and STI testing at one year. Which intervention components are effective will be explored by collecting data on the theoretical constructs on the pathway to behaviour change influenced by the intervention components. The cost-effectiveness of the intervention will be established.

METHODS AND ANALYSIS

Design and setting

Safetxt is a single blind parallel group randomised superiority trial with a 1:1 allocation ratio designed to establish the effects of a safer sex intervention delivered by text message on the cumulative incidence of chlamydia and gonorrhoea infection at one year. (See Figure 1 for the trial flow chart).

Potential participants testing positive for chlamydia, gonorrhoea or diagnosed with non-specific urethritis (NSU) will be identified from UK STI testing services by research staff based at the service (a full list of participating services is available at http://safetxt.lshtm.ac.uk/participating-sites/). The intervention is delivered by text message in the community. Trial recruitment started on the 1st April 2016. Completion of follow up and closure of the data set is planned for early spring 2020.

Characteristics of participants

For peer review only - http://bmjopen.bmj.com/site/about/guidelines.xhtml

Inclusion criteria

Participants will be eligible if they are between 16 and 24 years of age, own a personal mobile phone, are able to provide informed consent and who, in the 2 weeks prior to their recruitment, have either been diagnosed with chlamydia, gonorrhoea, or NSU, or have started treatment for chlamydia, gonorrhoea or NSU.

Exclusion criteria

Participants will be excluded if they are known to be a sexual partner of someone already recruited to the trial.

Recruitment and consent

Research staff based at STI testing services will identify potential participants. They will provide potential participants with trial information at one of three time points, i.e. when potential participants (i) attend the service and are diagnosed with chlamydia, gonorrhoea or NSU; (ii) receive positive test results for chlamydia or gonorrhoea by phone; or (iii) collect treatment for chlamydia, gonorrhoea or NSU from services.

Research staff will provide potential participants with verbal and written information (Supplementary file 1) about the trial and/or participants will view trial information on the website. Participants will be able to join the trial by either providing informed written consent to the research staff, or by providing consent online (Supplementary file 2) at the trial website or site staff can ask eligible participants if they are happy for their details to be given to research assistants based at the London School of Hygiene & Tropical Medicine Clinical Trials Unit (LSHTM CTU). In this case, LSHTM CTU will contact participants to recruit to the trial. Strategies for achieving adequate enrolment include site visits, training, feedback and rewards, sharing successful recruiting strategies and newsletters.

Allocation and blinding

Participants will be randomly allocated (1:1 ratio), using a computer-based randomisation system, to a safer sex intervention delivered by text messaging, or to a control group, ensuring allocation concealment. Allocation concealment is ensured as the randomisation system is independent, automated and remote.

An electronic link to the computer-based randomisation programme will result in the generation of a research number and allocation to the intervention or control group. The system will then automatically deliver intervention or control group texts according to the allocation.

For peer review only - http://bmjopen.bmj.com/site/about/guidelines.xhtml

Given the nature of the intervention, participants will be aware of their assigned group. Research assistants entering data, laboratory staff and researchers conducting the final analysis are masked to allocation until after the analysis is complete.

Interventions:

Safetxt Intervention group

The intervention aims to increase safer sex in three ways: i) encouraging participants to correctly follow STI treatment instructions including informing partner(s) about infection; ii) promoting condom use with new or casual partners; and iii) encouraging participants to obtain testing for STI prior to unprotected sex. Participants in the intervention group will receive regular messages delivered by text message in community settings according to a predetermined schedule. The intervention was informed by the capability, opportunity and motivation model of behaviour (COM-B)(28). It aims to influence the knowledge, beliefs, self-efficacy, skills, social and interpersonal influences that have important effects on motivation, capability and opportunity to reduce sexual risk behaviour.

Over the first ten days participants are sent messages targeting engagement with the intervention, taking treatment, avoiding sex for 7 days after treatment and telling partner (s) about an infection. These messages provide non-judgemental, non-stigmatising information about STI. They provide suggestions about when, where, and how to tell partner(s) about an infection and examples of how others told partners covering a range of different types of relationship (e.g. casual, long term).

Messages then target condom use and testing for STIs before having sex without a condom with a new partner. Topics cover risk assessment, instructions on how to use condoms, positive aspects of condom use, tips on preventing condom problems and examples of how others resolved condom use problems. Participants are prompted to think about their own success in achieving safer sex strategies, risks they had taken and what they could do differently in the future. Messages included advice regarding testing before unprotected sex with a new partner. Participants are sent links to: services; support for those concerned about partner violence; and web-based information regarding contraception, alcohol and sexual risk, how to use a condom and general communication about sex. The messages provide social support for safer sex behaviours and acknowledge participants experiences.

The intervention employs educational, enabling and incentivising behaviour change functions and twelve behaviour change techniques: information about health consequences of behaviour;

For peer review only - http://bmjopen.bmj.com/site/about/guidelines.xhtml

instruction on how to carry out the behaviour; demonstrations of risk reduction behaviour; social support; emotional support; social rewards; non-specific incentives; encouragement to add objects to the environment; anticipated regret; problem solving; action planning techniques; and reframing (29) . The information on safer sexual practices is in accordance with existing guidelines.

The messages are tailored according to gender and sexual orientation. WSM, MSM, MSMW, WSMW are sent messages about how others had negotiated condom use. WSM and MSW are sent messages about emergency contraception. MSM and MSMW are sent messages about post exposure prophylaxis (PEP). WSW only are not sent messages about condom use. The information provided is specific to the STI diagnosed. This tailoring results in different numbers of messages being sent to those of different gender and sexual orientation.

The core message sets include: 74 messages for women who have sex with men (WSM) and women who had sex with men and women (WSMW); 42 messages for women who have sex only with women (WSW); 69 messages for men who have sex with women (MSW) and 79 messages for men who have sex with men and women (MSMW); and 76 messages for men who have sex with men (MSM). Recipients can request additional messages on specific topics. Participants are sent: 4 messages per day for days 1-3, then 1-2 messages per day for days 4-28, then 2-3 messages per week for month 2, and 2-5 messages per month for months 3-12

Control group

Participants in the control group will receive a monthly untailored text message asking for information about changes in postal or email addresses. Example control group message: 'Thank you for taking part in the texting study. Remember to let us know if your contact details have changed by replying to this text or emailing safetxt@lshtm.ac.uk'

All participants will receive usual care and will be free to seek any other existing service or support they wish.

All messages are sent automatically from a large database to an aggregator, which conveys messages to each participant in the community via their network. The success of delivery of messages at each step is monitored by the aggregator and computer system that generates the messages. A member of the trial team will automatically be notified if there is any failure in the delivery of messages. All participants will be able to set embargoed times when they do not want

For peer review only - http://bmjopen.bmj.com/site/about/guidelines.xhtml

to receive messages. Participants will be able to stop text messages, but continue with the trial follow up, by texting 'stop' to the short code number.

Outcomes

Our primary outcome will be the cumulative incidence of chlamydia and gonorrhoea infection at one year assessed by nucleic acid amplification tests: urine for men (with additional pharyngeal and anal swabs for MSM) and self-taken vulvo-vaginal swab for women.

Secondary outcomes

Secondary outcomes at four weeks

Behaviours

- Whether participants took the (prescribed antibiotic) treatment and avoided sex for 7 days after treatment
- Whether they told the last person they had sex with before testing positive, that they needed to get treatment
- Clinic attendance by partner for treatment (identified from clinic records)
- Condom use at last sex

Process outcomes – scores of the theoretical constructs underlying the components of the intervention (behaviour change mediators):

- Attitudes towards partner notification
- Self–efficacy in telling a partner about an infection
- Self–efficacy in negotiating condom use
- Correct condom use self-efficacy
- Knowledge related to STIs

Secondary outcomes at one year

STIs

- Diagnosed with any STI after joining the trial according to self-report confirmed by postal test results and clinic records

Behaviours

- Condom use at last sex
- Number of sexual partners since joining the trial
- Sex with someone new since joining the trial
- Condom use at first sex with most recent new partner

For peer review only - http://bmjopen.bmj.com/site/about/guidelines.xhtml

- Self-reported sexually transmitted infection testing for self - prior to first sex with most recent new partner
- Sexually transmitted infection testing for self - prior to first sex with most recent new partner (confirmed according to clinic record that testing occurred)
- Participant's report as to whether their most recent new partner was tested for sexually transmitted infection prior to sex with them
- Number of sexual partners since joining the trial
- Reading and sharing of intervention content
- Number of text messages read
- Whether anyone else read the messages, if yes how did the participant feel about them reading the messages?
- Reading someone else's messages in the trial (to be reported for control group participants)
- Someone else in the trial reading participants' messages (to be reported for intervention group participants)

Potential harms

- Car accident where the participant was the driver in the past year
- Experience of partner violence in the past year

Data collection

The primary outcome will be assessed using chlamydia and gonorrhoea tests collected by post at one year and clinic records of completed tests. STI test kits for chlamydia and gonorrhoea will be posted (in P650 standard packaging) to respondents. Directions in the pack will ask participants to provide a vaginal swab (women), a urine sample (men), and additional anal and pharyngeal samples (men who have sex with men) and post it in the prepaid and addressed envelope to the laboratory. Test kits will be identified by lab number only, rendering the laboratory staff blind to the participant's allocation. The accredited laboratory will destroy samples after testing is completed.

Results of the STI testing will be reported on the secure trial lab site by lab code only. Clinic records will be checked to identify participants who have had a positive STI diagnosis since joining the study, confirm self-reported STI testing and partner attendance for treatment.

For peer review only - http://bmjopen.bmj.com/site/about/guidelines.xhtml

For peer review only

At 4 weeks and at one-year, postal questionnaires will be sent to all participants to collected self-reported data. Non-responders will be contacted by any method the participant agrees to at enrolment (post, email, text message, telephone call). Participants can directly enter self-reported outcome data via a web-based data entry form. Paper based self-reported outcome data or self-reported data collected by telephone will be directly entered into the web-based data entry form by a trial assistant blinded to treatment allocation.

Participants will be sent a £5 unconditional incentive with each postal request i.e. when sending the 4 weeks questionnaire and 1-year test and questionnaire. Participants who return the test sample will be sent £20. See Figure 2 for the Schedule of enrolment, interventions and assessments. All outcome data will be collected on participants who choose to stop messages unless they withdraw from the trial.

Data management

Anonymised research data will be held on a secure system and password protected. All data systems will be set up with checks to alert the trial assistants if data being entered are illogical, inconsistent or incomplete.

Personal details will be stored on a password protected computer held on a secure server at the LSHTM. In accordance with the Data Protection Act of 1998 (30), this information will be stored separately from any anonymised research data, and will be deleted at the end of the study.

Any paper-based data will be locked in a cabinet within a room. All trial procedures are in accordance with the principles of Good Clinical Practice. Essential documents of the sponsor/trial organisers and investigators will be retained for at least ten years after completion of the trial.

Sample size

The study is powered for the primary outcome measure of cumulative incidence of chlamydia and/or gonorrhoea infection at one year. Two main factors determine the number of participants needed for this trial: the estimated event rate, and the size of the treatment effect. Our estimates are based on the following data:

Estimated event rate

The estimated event rate for the cumulative incidence of STI at 1 year is 20%, based on the event rate in cohort studies and the pilot trial (3).

For peer review only - http://bmjopen.bmj.com/site/about/guidelines.xhtml

Size of treatment effect

Because the intervention can be administered to large populations at low cost, even a modest reduction in treatable STIs would be worthwhile. The trial has therefore been designed to detect a reduction in chlamydia or gonorrhoea infection of 20% versus 16% (RR 0.8), which is similar to the effects of face-to-face safer sex interventions (7).

Sample size calculation

In the pilot trial 2% of the control group viewed messages delivered to intervention participants. If the real difference in STI infection at one year follow up is 20% versus 16% then with contamination of 2% the trial would detect a difference of 19.9% vs 16%. (Calculated based on 2% of the control group having an infection rate the same as the intervention group = (98% x 20%) + (2% x16%) = 19.9%).

Five thousand participants would provide 90% power to detect this difference, allowing for 20% losses to follow-up.

The TSC reviewed the blinded event rate after 546 patients had completed 12 months follow up and recommended an increase in the sample size to 6250 due to a lower than expected event rate of 15.6%.

Analysis

A detailed statistical analysis plan (SAP) will be finalised and submitted to the TSC before the end of the trial and unblinding. For the primary outcome and other binary outcomes, we will estimate any differences between the groups using logistic regression and we will report odds ratios with 95% confidence intervals and p-values, adjusting for key baseline predictors of outcome. These will specified in advance in the SAP. For continuous outcomes, we will use linear regression to test for a difference in mean scores between the arms. All analyses will be based on the intention-to-treat principle.

For the primary analysis Multiple Imputation by Chained Equations (MICE) will be used in order to account for missing data (31). MICE makes appropriate assumptions for accommodating missing data in the analysis based on the predictors of outcome and the predictors of loss to follow up. MICE is recognised as a way of reducing bias and increasing precision of trial results and is increasingly used as the primary analysis in randomised controlled trials (12, 31). One hundred imputed datasets will be generated and the point estimates and standard errors will be combined using Rubin's rules. We will perform information-anchored sensitivity analysis using the delta-method (32).

For peer review only - http://bmjopen.bmj.com/site/about/guidelines.xhtml

Supplementary analyses.

Before fitting our primary analysis model, we need to identify appropriate auxiliary variables to include in the imputation model. Such auxiliary variables should have information about the missing outcome. Specifically, an independent statistician will use a dataset without the treatment variable and perform regression analyses to identify key predictors of outcomes and which combination of baseline variables and time to negative STI test in clinic should be included as auxiliary variables in the imputation model. We will supplement this by performing and reporting a complete case analysis (where any participants with missing information on any covariate or outcome shall be excluded).

Sub group analyses

A limited number of subgroup analyses will be undertaken adjusting for other key predictors of outcome. Subgroup analyses include: age (16-19, 20-24), sexual orientation (men who have sex with men or men and women, men who have sex only with women, women who have sex with men or men and women, women who only have sex with women), ethnic group (Caucasian, Black, other) and age at which left education (16 or under, 17 or over). The subgroups will be analysed by inclusion of an interaction term between treatment group and the subgroup in the appropriate regression model for the outcome. Data will be presented by categories of subgroup using effect estimates and 99% confidence intervals. For each subgroup analysis, missing outcomes will be imputed consistent with the hypothesis of an interaction (33) .

Process outcomes

Candidate questionnaire items were identified for the measurement of the following constructs: attitudes towards partner notification; self–efficacy in telling a partner about an infection; self–efficacy in negotiating condom use; correct condom use self-efficacy; knowledge related to STIs. At four weeks, each of the five process outcomes were measured using the candidate questionnaire items (3 to 5 items per process outcome). Based on these items, we will carry out confirmatory factor analysis (CFA) to refine and identify a valid measurement model. For the main trial analyses, the final measurement model will be combined with the intervention group allocation, along with key baseline predictors, extending the measurement model to a Multiple Causes Multiple Indicators (MIMIC) model. This model will be used to estimate the effects of the intervention on the five hypothesised process outcomes.

Economic evaluation

The economic modelling required to assess the cost-effectiveness of the intervention will estimate the annual probability of members of the target group acquiring chlamydia, gonorrhoea

For peer review only - http://bmjopen.bmj.com/site/about/guidelines.xhtml

and NSU with and without the intervention (based on the experience of those in the control and intervention arms of the trial). Detailed information will be collected on the costs of delivering the intervention. Secondary sources will be used to estimate the future NHS costs avoided as a consequence of avoided infections.

Patient involvement

Patients were involved in the development of the intervention (15) and the design of the trial materials and follow-up procedures (34). A patient of also a member of the Trial Steering Committee. Young people will be involved in the dissemination of the trial results.

Monitoring

The safetxt trial will not have a separate Data Monitoring Committee (DMC). No interim analyses of intervention effects will be conducted since the trial is of a behavioural support intervention unlikely to cause harm, and therefore, there are no stopping rules. Analysis of intervention effects will be conducted once at the end of the trial. The sponsor may audit the trial per their own risk assessment and schedule.

Harms will be assessed by self-reported data. Car accidents are the only demonstrated harm resulting from text messaging, hence the intention to collect data regarding involvement in road traffic accidents. The safetxt intervention aims to increase partner notification of STI status. Fear of partner violence has been reported to be a barrier to partner notification and partner notification has been identified as a factor which may trigger partner violence. However, no randomised controlled trials targeting increased partner notification detected a difference in partner violence between the control intervention groups. Data will be collected regarding the experience of partner violence at 12 months.

In addition, participants will be asked an open-ended question in the 12 month questionnaire: "Did anything good or bad happen as a result of being involved in the study or receiving the text messages? Please describe." The Clinical Trials Unit will keep detailed records of all adverse events reported. Reports will be reviewed by the Principal Investigator and the TSC to consider intensity, causality and expectedness. As appropriate, these will be reported to the sponsor and the Ethics Committee.

Ethics and Dissemination

Ethical approval for this trial was provided by the NHS Health Research Authority - London – Riverside Research Ethics Committee (REC reference: 15/LO/1665) and the LSHTM. All trial

For peer review only - http://bmjopen.bmj.com/site/about/guidelines.xhtml

participants will provide written informed consent prior to enrolment in the trial. Participants' can withdraw from the trial on request. Participant will receive usual NHS care there is no ancillary care.

Any modifications to the protocol will be approved by the ethics committee prior to implementation. Records of any important modifications will be submitted as an addendum the journal in which this protocol is published.

Trial results will be published open-access in peer-review journals. Authorship will be on the basis of meeting the criteria recommended by the International Committee of Medical Journal Editors (ICMJE).

After the publication of our main trial findings, the anonymised trial dataset will be available on request from the corresponding author.

DISCUSSION

STIs such as chlamydia and gonorrhoea confer a heavy burden of disease among young people with long-term sequelae such as infertility. Safer sex behaviours such as condom use, notifying partner(s) about an existing STI and STI testing reduce the risk of STI, but young people may lack the knowledge, confidence and skills needed to adopt these behaviours. Safer sex interventions delivered by mobile phone are acceptable to young people and show promise in increasing safer sex behaviours, but to date their effects on STI are not reliably known.

If it proves to be effective, this low-cost intervention could have an important impact in improving sexual health and reducing inequalities among young people in the UK. There is likely to be international interest in the impact of the intervention as short written messages delivered via mobile phones are increasingly used for behavioural support worldwide and sexually transmitted infections remain an important cause of morbidity and mortality.

For peer review only - http://bmjopen.bmj.com/site/about/guidelines.xhtml

Supplementary files

Supplementary File 1. Patient Information sheet

Supplementary File 2. Patient consent form

List of abbreviations

CTU – Clinical Trials Unit

GCP – Good Clinical Practice STI - Sexually transmitted infection

HIV – Human Immunodeficiency Virus

LSHTM - London School of Hygiene & Tropical Medicine

MICE – Multiple Imputation by Chained Equations

MSM – Men who have sex with men

NAAT - Nucleic Acid Amplification Test

NSU – Non-Specific Urethritis

REFERENCES

1. Sonnenberg P, Clifton S, Beddows S, Field N, Soldan K, Tanton C, et al. Prevalence, risk factors, and uptake of interventions for sexually transmitted infections in Britain: findings from the National Surveys of Sexual Attitudes and Lifestyles (Natsal). Lancet. 2013;382(9907):1795-806.
2. Cates W, Jr., Wasserheit JN. Genital chlamydial infections: epidemiology and reproductive sequelae. Am J Obstet Gynecol. 1991;164(6 Pt 2):1771-81.
3. Hosenfeld CB, Workowski KA, Berman S, Zaidi A, Dyson J, Mosure D, et al. Repeat infection with Chlamydia and gonorrhea among females: a systematic review of the literature. Sex Transm Dis. 2009;36(8):478-89.
4. Sheringham J, Mann S, Simms I, Stafford M, Hart GJ, Raine R. It matters what you measure: a systematic literature review examining whether young people in poorer socioeconomic circumstances are more at risk of chlamydia. Sexually Transmitted Infections. 2013;89(2):175-80.
5. De Waroux OLP, Harris R, Hughes G, Crook P. The epidemiology of gonorrhoea in London: a Bayesian spatial modelling approach. Epidemiology and infection. 2014;142(01):211-20.
6. Hughes G, Nichols T, Peters L, Bell G, Leong G, Kinghorn G. Repeat infection with gonorrhoea in Sheffield, UK: predictable and preventable? Sexually Transmitted Infections. 2012.
7. Free C, Roberts IG, Abramsky T, Fitzgerald M, Wensley F. A systematic review of randomised controlled trials of interventions promoting effective condom use. J Epidemiol Community Health. 2011;65(2):100-10.

For peer review only - http://bmjopen.bmj.com/site/about/guidelines.xhtml

8. French RS, Bonell C, Wellings K, Weatherburn P. An exploratory review of HIV prevention mass media campaigns targeting men who have sex with men. BMC Public Health. 2014;14:616.
9. Estcourt C SL, Mercer CH, Copas A, Muniia P, Rait G, et al. Can accelerated partner therapy (APT) improve outcomes of partner notification for women diagnosed with genital chlamydia in primary care settings: A pilot randomized controlled trial in general practice and community sexual health services. Sexually Transmitted Diseases Conference; Conference start: 20140609 Conference end: 20140612; Atlanta, GA United States2014. p. S32-S3.
10. Control ECfDPa. Public health benefits of partner notification for sexually transmitted infections and HIV. Stockholm: European Centre for Disease Prevention and Control: 2013.
11. Guerriero C, Cairns J, Roberts I, Rodgers A, Whittaker R, Free C. The cost-effectiveness of smoking cessation support delivered by mobile phone text messaging: Txt2stop. Eur J Health Econ. 2013;14(5):789-97.
12. Free C, Knight R, Robertson S, Whittaker R, Edwards P, Zhou W, et al. Smoking cessation support delivered via mobile phone text messaging (txt2stop): a single-blind, randomised trial. Lancet. 2011;378(9785):49-55.
13. Free C, Phillips G, Galli L, Watson L, Felix L, Edwards P, et al. The effectiveness of mobile-health technology-based health behaviour change or disease management interventions for health care consumers: a systematic review. PLoS Med. 2013;10(1):e1001362.
14. Burns K, Keating P, Free C. A Systematic Review of mHealth RCTs for Sexual Health. BMC Public Health. 2016;16:778.
15. Free C, McCarthy O, French R, Wellings K, Michie S, Roberts I, et al. Can text messages increase safer sex behaviours in young people? Intervention development and pilot randomised controlled trial. Health Technol Assess 2016;20(57).
16. de Tolly K, Skinner D, Nembaware V, Benjamin P. Investigation into the use of short message services to expand uptake of human immunodeficiency virus testing, and whether content and dosage have impact. Telemed J E Health. 2012;18(1):18-23.
17. Gold J, Lim MS, Hellard ME, Hocking JS, Keogh L. What's in a message? Delivering sexual health promotion to young people in Australia via text messaging. BMC public health. 2010;10:792.
18. Lim MS, Hocking JS, Aitken CK, Fairley CK, Jordan L, Lewis JA, et al. Impact of text and email messaging on the sexual health of young people: a randomised controlled trial. J Epidemiol Community Health. 2012;66(1):69-74.
19. Field N, Mercer CH, Sonnenberg P, Tanton C, Clifton S, Mitchell KR, et al. Associations between health and sexual lifestyles in Britain: findings from the third National Survey of Sexual Attitudes and Lifestyles (Natsal-3). Lancet. 2013;382(9907):1830-44.
20. Downing SG, Cashman C, McNamee H, Penney D, Russell DB, Hellard ME. Increasing chlamydia test of re-infection rates using SMS reminders and incentives. Sex Transm Infect. 2013;89(1):16-9.
21. Mugo PM, Wahome EW, Gichuru EN, Mwashigadi GM, Thiong'o AN, Prins HA, et al. Effect of Text Message, Phone Call, and In-Person Appointment Reminders on Uptake of Repeat HIV Testing among Outpatients Screened for Acute HIV Infection in Kenya: A Randomized Controlled Trial. PLoS ONE [Electronic Resource]. 2016;11:e0153612.
22. Odeny TA, Bailey RC, Bukusi EA, Simoni JM, Tapia KA, Yuhas K, et al. Effect of text messaging to deter early resumption of sexual activity after male circumcision for HIV prevention: a randomized controlled trial. J Acquir Immune Defic Syndr. 2014;65(2):e50-7.
23. Suffoletto B, Akers A, McGinnis KA, Calabria J, Wiesenfeld HC, Clark DB. A sex risk reduction text-message program for young adult females discharged from the emergency department. J Adolesc Health. 2013;53(3):387-93.

For peer review only - http://bmjopen.bmj.com/site/about/guidelines.xhtml

24. Jones R, Hoover DR, Lacroix LJ. A randomized controlled trial of soap opera videos streamed to smartphones to reduce risk of sexually transmitted human immunodeficiency virus (HIV) in young urban African American women. Nurs Outlook. 2013;61(4):205-15 e3.
25. Thomas N, Murray E, Rogstad KE. Confidentiality is essential if young people are to access sexual health services. Int J STD AIDS. 2006;17(8):525-9.
26. French RS, McCarthy O, Baraitser P, Wellings K, Bailey JV, Free C. Young People's Views and Experiences of a Mobile Phone Texting Intervention to Promote Safer Sex Behavior. JMIR mHealth and uHealth. 2016;4(2).
27. McCarthy OL, French RS, Baraitser P, Roberts I, Rathod SD, Devries K, et al. Safetxt: a pilot randomised controlled trial of an intervention delivered by mobile phone to increase safer sex behaviours in young people. BMJ Open. 2016;6(12).
28. Michie S, M van Stralen M, West R. The behaviour change wheel: A new method for characterising and designing behaviour change interventions

2011;6:42.
29. Michie S, Wood C, Johnston M, Abraham C, Francis J, Hardeman W. Behaviour change techniques: the development and evaluation of a taxonomic method for reporting and describing behaviour change interventions. Health Technology Assessment. 2015;19(99):1-188.
30. Data Protection Act. 1998.
31. Sterne JA, White IR, Carlin JB, Spratt M, Royston P, Kenward MG, et al. Multiple imputation for missing data in epidemiological and clinical research: potential and pitfalls. BMJ. 2009;338:b2393.
32. Cro S, Carpenter J, Kenward MIasataa. Information anchored sensitivity analysis: theory and application. Journal of the Royal Statistical Society. 2018;182:623-45.
33. Carpenter JR, Kenward M. Multiple imputation and its application. Chichester: Wiley; 2013.
34. McCarthy O, French R, Roberts I, Free C. Simple steps to develop trial follow-up procedures. Trials. 2016;17(28).

Figures

Figure 1. Trial flow chart

Figure 2. Schedule of enrolment, interventions and assessments

Authors' contributions

CF conceived the study, contributed to the development of the intervention and statistical analysis plan, is a member of the Trial Management Group, and had overall responsibility for the design and conduct of the trial and writing the protocol.

OM contributed to the development of the intervention, contributed to the statistical analysis plan, is a member of the Trial Management Group, contributed to the trial design, the refinement of the study protocol and approved the manuscript.

For peer review only - http://bmjopen.bmj.com/site/about/guidelines.xhtml

MP contributed to the statistical analysis plan, is a member of the Trial Management Group, contributed to the process evaluation design, contributed to the trial design, the refinement of the study protocol and approved the manuscript.

RK is a member of the Trial Management Group, is are responsible for the day-to-day management of the trial, contributed to the trial design, the refinement of the study protocol and approved the manuscript.

PE is the trial statistician who provides general statistical oversight, contributed to the statistical analysis plan, contributed to the trial design, the refinement of the study protocol and approved the manuscript.

RF contributed to the development of the intervention, provided input on the trial outcome measures, contributed to the trial design, the refinement of the study protocol and approved the manuscript.

PB contributed to the development of the intervention, contributed to the trial design, the refinement of the study protocol and approved the manuscript.

FH adapted the intervention messages for men who have sex with men, contributed to the trial design, the refinement of the study protocol and approved the manuscript.

KW contributed to the development of the intervention, provided input on the trial outcome measures, contributed to the trial design, the refinement of the study protocol and approved the manuscript.

IR provided expertise on trial design, contributed to the refinement of the study protocol and approved the manuscript.

JB contributed to the development of the intervention, the trial design, the refinement of the study protocol and approved the manuscript.

GH provided fundamental intellectual input into the design of the trial, the refinement of the study protocol and approved the manuscript.

SM provided behavioural science expertise, which informed the development of the intervention, contributed to the trial design, the refinement of the study protocol and approved the manuscript.

For peer review only - http://bmjopen.bmj.com/site/about/guidelines.xhtml

TC provided statistical expertise regarding the supplementary and subgroup analyses, provided general statistical advice, contributed to the trial design, the refinement of the study protocol and approved the manuscript.

GP provided statistical expertise for the Generalised Latent Variable Modelling framework, contributed to the trial design, the refinement of the study protocol and approved the manuscript.

JC provided statistical expertise regarding multiple imputation, contributed to the trial design, the refinement of the study protocol and approved the manuscript..

KT leads the economic evaluation, contributed to the trial design, the refinement of the study protocol and approved the manuscript.

KD contributed to the development of the intervention, contributed to the trial design, the refinement of the study protocol and approved the manuscript.

KP is a member of the Trial Management Group, is are responsible for the day-to-day management of the trial, contributed to the trial design, the refinement of the study protocol and approved the manuscript.

Funding

This trial is funded by the National Institute for Health Research Public Health Research (NIHR PHR) Programme (project number 14/182/07). The funding body have no role in any aspect of the design and conduct of this study, or the decision to submit the report for publication.

Competing interests

The authors declare that they have no competing interests.

Ethics approval

Ethics approval for this trial was provided by the NHS Health Research Authority - London – Riverside Research Ethics Committee (REC reference: 15/LO/1665) and the LSHTM.

Trial Sponsor

London School of Hygiene & Tropical Medicine is the main research sponsor for this trial. The sponsor had no role in any aspect of the design and conduct of this study, or the decision to

For peer review only - http://bmjopen.bmj.com/site/about/guidelines.xhtml

submit the report for publication. For further information regarding the sponsorship conditions, please contact the Research Governance and Integrity Office:

London School of Hygiene & Tropical Medicine, Keppel Street, London, WC1E 7HT

Tel: +44 207 927 2626 Email: patricia.henley@lshtm.ac.uk

Trial Steering Committee

The Trial Steering Committee (TSC) may recommend changes to the overall trial management processes for which there is evidence the current process may compromise the conduct of the trial or the safety of participants. The members of the TSC are: Professor Pippa Oakeshott (Chair), Dr Andrew Copas, Professor Michael Ussher, Mr Colum McGrady, Dr Michael Brady, and Professor Caroline Free.

Word count 3997

For peer review only

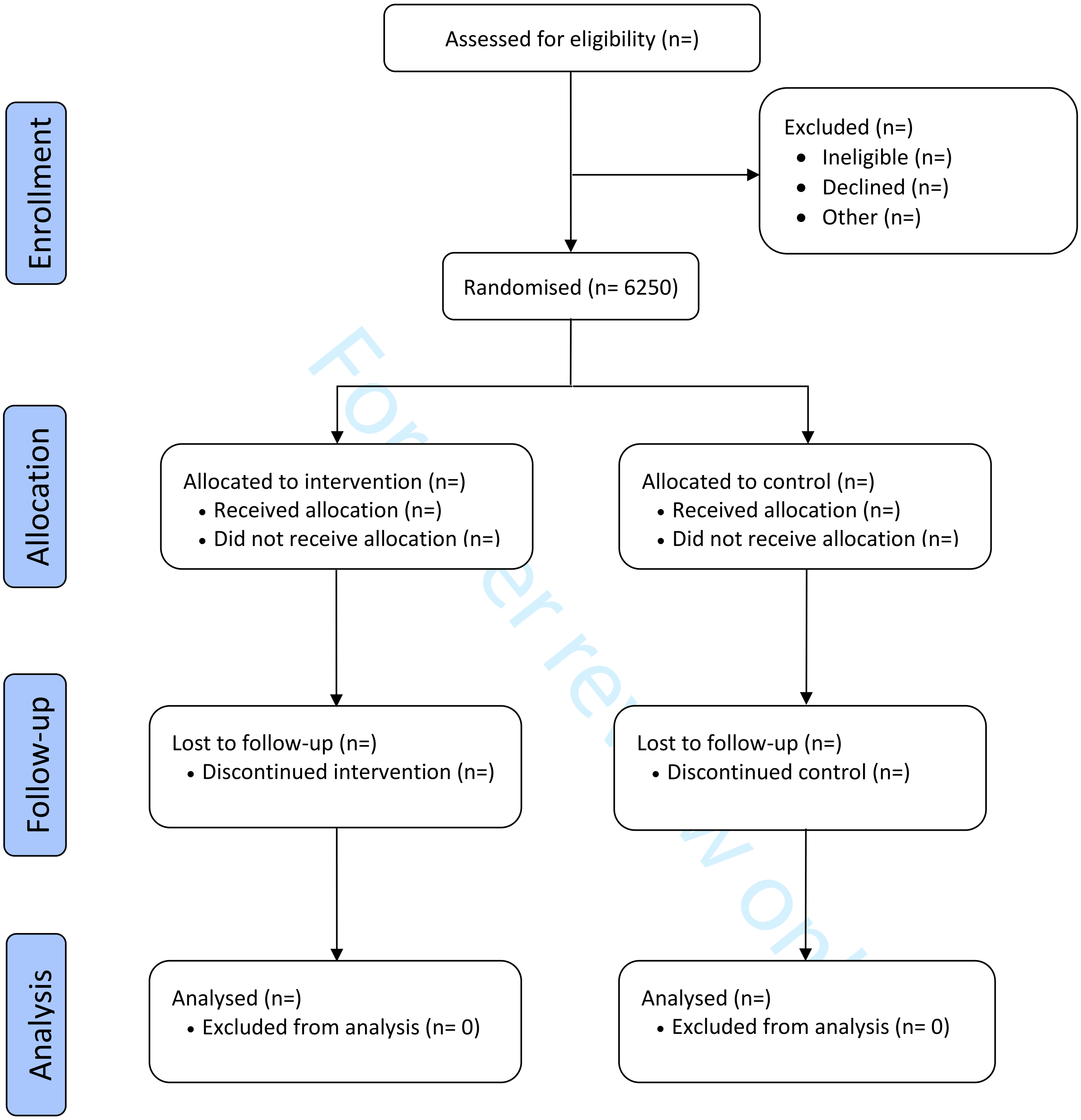
Enrollment
Assessed for eligibility (n=)
Excluded (n=)
• Ineligible (n=)
• Declined (n=)
• Other (n=)
Randomised (n= 6250)
Allocation
Allocated to intervention (n=)
• Received allocation (n=)
• Did not receive allocation (n=)
Allocated to control (n=)
• Received allocation (n=)
• Did not receive allocation (n=)
Follow-up
Lost to follow-up (n=)
• Discontinued intervention (n=)
Lost to follow-up (n=)
• Discontinued control (n=)
Analysis
Analysed (n=)
• Excluded from analysis (n= 0)
Analysed (n=)
• Excluded from analysis (n= 0)

Figure 2. Schedule of enrolment, interventions, and assessments.

	STUDY PERIOD				
	**Enrolment**	**Allocation**	**Post-allocation**		**Close-out**
**TIMEPOINT****	**0**	**0**	***Wk 4***	***Wk 52***	***Wk 76***
**ENROLMENT:**					
**Eligibility screen**	X				
**Informed consent**	X				
**Allocation**		X			
**INTERVENTIONS:**					
safetxt text messages		◆———	———	———◆	
control text messages		◆———	———	———◆	
**ASSESSMENTS:**					
Socio-demographic characteristics	X				
Recent sexual behaviour	X		X	X	X
STI diagnosis	X				
Correct treatment of STI			X		
Partner notification			X		
Attitudes, self-efficacy, knowledge			X		
STI test (postal NAAT)				X	X
STI testing prior to sex with new partner				X	X
Number of text messages read				X	X
Anyone else read text messages				X	X
Experience of car accident				X	X
Experience of partner violence				X	X

*

For peer review only

A randomised controlled trial of a safer sex intervention delivered through mobile phone messaging

Local site logo

Participant's Information Sheet
safetxt: a randomised controlled trial of a safer sex intervention

We are inviting you to take part in a research study. Before you decide, it is important that you know why we are doing the study and what is involved. Please read the following information carefully.

What is the study?
This study is testing whether text messages providing information and tips to increase safer sex helps young people adopt safer sex behaviours.

Why have I been chosen?
You are aged 16-24 and have recently received a positive test result for Chlamydia, gonorrhoea or had a non specific urethritis diagnosis.

Do I have to take part?
No, it is up to you to decide whether to take part.

What will happen if I take part?
After you have had all your questions answered and have agreed to take part, we will ask you to complete a consent form. You can do this on our secure and confidential study website, by filling out a paper version, by text message or by email. We will then ask you to complete a short confidential questionnaire providing details about yourself.

After you have completed the questionnaire, **an automated computer system will put you into one of two groups by chance (randomly)**. One group will receive safer sex text messages for 1-2 weeks, then about 5 messages a month for 11 months. The other group will receive text messages asking you to confirm your contact details. These messages will not be about sexual health. This group will receive one message a month for 12 months.

We will ask you to complete the questionnaire again at 1 and 12 months after signing up. This is to see how things have been for you after joining the study. Also at 12 months we will ask you to provide a sample to test for Chlamydia and gonorrhoea. The testing kit will be sent to you by post. **Participating in the study involves returning one urine sample or self-taken swab to test for Chlamydia and gonorrhoea.**

Will you compensate me for the time this takes?
We will offer you £5 as compensation for your time at each follow up: for the 1 month questionnaire and the 12 month questionnaire. You will be compensated for your time for returning your 12 month sample.

We do not charge for text messages we send to you.

What are the alternatives?
You do not have to take part.

What are the possible disadvantages in taking part?
Completing the questionnaires and providing a sample will take up some of your time. It is possible that the messages we send could be read by someone else. If you are concerned about this, you can password-lock your phone and delete the messages after you read them.

For peer review only - http://bmjopen.bmj.com/site/about/guidelines.xhtml

What are the possible benefits of taking part?

We cannot promise that your health will benefit from taking part but you may find the messages helpful and you may learn about safer sex behaviours.

What happens when this study stops?

When the study stops, we will look at the data collected to see if the text messages helped increase safer sex behaviours.

What will happen if I don't want to carry on with the study?

You can stop receiving text messages by texting STOP or pause the text messages by texting PAUSE. You can withdraw at any time by letting the Clinical Trials Unit know (safetxt@LSHTM.ac.uk or freephone 0800 008 7197). You do not have to give a reason for wanting to withdraw.

What if there is a problem?

You can contact the Clinical Trials Unit if there is a problem. If you would like to make a formal complaint, contact Dr Caroline Free who will follow the complaints procedure.

Will my taking part in this study be kept confidential?

Yes. Your answers to the questionnaires will be stored anonymously and your contact details and Chlamydia and gonorrhoea test will be kept confidential. You will be allocated a unique study number when you enrol in the study. We will not inform your parents about your involvement in this research. If you were tested for sexually transmitted infections during the study, we will ask your clinic or GP for your test results. We will also ask for information on whether or not a partner was treated. To keep the messages confidential, password-lock your phone and delete messages after you read them.

Your records will be made available to people authorised to work on the study. They may also need to be made available to people for ensuring that the study is carried our correctly.

With your permission, we will ask your clinic or GP for your test results for sexually transmitted infections after you have finished participating in the study. This is to allow the study researchers to look into long term effects about your sexual health.

What will happen to the samples that I give?

You will post your sample directly to a laboratory to test for Chlamydia and gonorrhoea infection. The laboratory will destroy the samples after they have finished testing. You will be notified if the test shows that there is Chlamydia or gonorrhoea infection. The clinic where you signed up to the study will contact you to arrange treatment.

How long will your data be stored for?

Your data will be kept for 10 years after the trial is completed. However, in accordance with the Data Protection Act of 1998, any personal identifiable data will not be kept longer than necessary and will be deleted within 3 months after you have finished the study.

What will happen to the results of the research study?

The results will be published in a scientific journal so that other people know about it. If you would like a copy of the results please contact the Clinical Trials Unit.

If you want to receive the safer sex messages after your participation in the study, please let us know and we will send them to you.

If the results of the study show that the messages have helped, they will be made available to all young people.

Who is organising and funding the research?

The study is run by Dr Caroline Free (Chief Investigator) from the London School of Hygiene and Tropical Medicine, at the University of London. The UK National Institute for Health Research is funding the trial.

For peer review only - http://bmjopen.bmj.com/site/about/guidelines.xhtml

Who has reviewed the trial?

London Riverside NHS Medical Research Ethics Committee.

Thank you for taking the time to consider taking part.

If you would like further information please contact the local Research Nurse or Clinical Trials Unit (details below)

The team contact details:

INSERT LOCAL CONTACT DETAILS

If you have any questions about the trial, please email: safetxt@LSHTM.ac.uk or Freephone: 0800 008 7197

For peer review only - http://bmjopen.bmj.com/site/about/guidelines.xhtml

safetxt

A randomised controlled trial of a safer sex intervention delivered through mobile phone messaging

Hospital logo to be inserted

Consent Form

Chief Investigator: Dr Caroline Free

Name of local researcher: *(Please insert local details)*

Please **initial box**

I confirm that I have read and understand the information sheet [version 8, 16th April 2018] for the above study. I have had the opportunity to consider the information, ask questions and have had these answered satisfactorily.

I understand that participation in this study is my choice and that I am free to withdraw at any time without giving any reason, without my medical care or legal rights being affected.

I understand that I should not read or send text messages while driving a vehicle.

I understand that relevant sections of data collected during the study may be looked at by the NHS Trust or LSHTM, where it is relevant to my taking part in this research. I give permission for these individuals to have access to my records.

I agree to the study researchers letting my clinic know of any positive test results I receive so they can arrange treatment.

I agree that my clinic/GP can provide the researchers with my test results for sexually transmitted infections.

If I give my partner(s) details to my clinic, I agree to the researchers being told if they have been tested.

I understand that all information I provide will remain confidential in accordance with the Data Protection Act of 1998.

I understand that the information collected about me will be used to support other research in the future, and may be shared anonymously with other researchers.

I agree to take part in the above study.

Optional

After the study, I agree that my clinic/GP can provide the researchers with my sexually transmitted infection results.

Name of Patient	Date and time	Signature
Name of researcher taking consent	Date and time	Signature

For peer review only - http://bmjopen.bmj.com/site/about/guidelines.xhtml

SPIRIT 2013 Checklist: Recommended items to address in a clinical trial protocol and related documents*

Section/item	Item No	Description	Addressed on page number
**Administrative information**			
Title	1	Descriptive title identifying the study design, population, interventions, and, if applicable, trial acronym	1
Trial registration	2a	Trial identifier and registry name. If not yet registered, name of intended registry	3
	2b	All items from the World Health Organization Trial Registration Data Set	3
Protocol version	3	Date and version identifier	3
Funding	4	Sources and types of financial, material, and other support	18
Roles and responsibilities	5a	Names, affiliations, and roles of protocol contributors	18
	5b	Name and contact information for the trial sponsor	19
	5c	Role of study sponsor and funders, if any, in study design; collection, management, analysis, and interpretation of data; writing of the report; and the decision to submit the report for publication, including whether they will have ultimate authority over any of these activities	19
	5d	Composition, roles, and responsibilities of the coordinating centre, steering committee, endpoint adjudication committee, data management team, and other individuals or groups overseeing the trial, if applicable (see Item 21a for data monitoring committee)	19

For peer review only

Introduction

Background and rationale	6a	Description of research question and justification for undertaking the trial, including summary of relevant studies (published and unpublished) examining benefits and harms for each intervention	5-6
	6b	Explanation for choice of comparators	8-9
Objectives	7	Specific objectives or hypotheses	6
Trial design	8	Description of trial design including type of trial (eg, parallel group, crossover, factorial, single group), allocation ratio, and framework (eg, superiority, equivalence, noninferiority, exploratory)	6

Methods: Participants, interventions, and outcomes

Study setting	9	Description of study settings (eg, community clinic, academic hospital) and list of countries where data will be collected. Reference to where list of study sites can be obtained	6
Eligibility criteria	10	Inclusion and exclusion criteria for participants. If applicable, eligibility criteria for study centres and individuals who will perform the interventions (eg, surgeons, psychotherapists)	6-7
Interventions	11a	Interventions for each group with sufficient detail to allow replication, including how and when they will be administered	8-9
	11b	Criteria for discontinuing or modifying allocated interventions for a given trial participant (eg, drug dose change in response to harms, participant request, or improving/worsening disease)	14
	11c	Strategies to improve adherence to intervention protocols, and any procedures for monitoring adherence (eg, drug tablet return, laboratory tests)	n/a
	11d	Relevant concomitant care and interventions that are permitted or prohibited during the trial	9
Outcomes	12	Primary, secondary, and other outcomes, including the specific measurement variable (eg, systolic blood pressure), analysis metric (eg, change from baseline, final value, time to event), method of aggregation (eg, median, proportion), and time point for each outcome. Explanation of the clinical relevance of chosen efficacy and harm outcomes is strongly recommended	9-10
Participant timeline	13	Time schedule of enrolment, interventions (including any run-ins and washouts), assessments, and visits for participants. A schematic diagram is highly recommended (see Figure)	Figure 2

For peer review only - http://bmjopen.bmj.com/site/about/guidelines.xhtml

Sample size	14	Estimated number of participants needed to achieve study objectives and how it was determined, including clinical and statistical assumptions supporting any sample size calculations	12
Recruitment	15	Strategies for achieving adequate participant enrolment to reach target sample size	7

Methods: Assignment of interventions (for controlled trials)

Allocation:

Sequence generation	16a	Method of generating the allocation sequence (eg, computer-generated random numbers), and list of any factors for stratification. To reduce predictability of a random sequence, details of any planned restriction (eg, blocking) should be provided in a separate document that is unavailable to those who enrol participants or assign interventions	7
Allocation concealment mechanism	16b	Mechanism of implementing the allocation sequence (eg, central telephone; sequentially numbered, opaque, sealed envelopes), describing any steps to conceal the sequence until interventions are assigned	7
Implementation	16c	Who will generate the allocation sequence, who will enrol participants, and who will assign participants to interventions	7
Blinding (masking)	17a	Who will be blinded after assignment to interventions (eg, trial participants, care providers, outcome assessors, data analysts), and how	7
	17b	If blinded, circumstances under which unblinding is permissible, and procedure for revealing a participant's allocated intervention during the trial	n/a

Methods: Data collection, management, and analysis

Data collection methods	18a	Plans for assessment and collection of outcome, baseline, and other trial data, including any related processes to promote data quality (eg, duplicate measurements, training of assessors) and a description of study instruments (eg, questionnaires, laboratory tests) along with their reliability and validity, if known. Reference to where data collection forms can be found, if not in the protocol	11
	18b	Plans to promote participant retention and complete follow-up, including list of any outcome data to be collected for participants who discontinue or deviate from intervention protocols	11

For peer review only - http://bmjopen.bmj.com/site/about/guidelines.xhtml

Data management	19	Plans for data entry, coding, security, and storage, including any related processes to promote data quality (eg, double data entry; range checks for data values). Reference to where details of data management procedures can be found, if not in the protocol	11-12
Statistical methods	20a	Statistical methods for analysing primary and secondary outcomes. Reference to where other details of the statistical analysis plan can be found, if not in the protocol	12-14
	20b	Methods for any additional analyses (eg, subgroup and adjusted analyses)	13
	20c	Definition of analysis population relating to protocol non-adherence (eg, as randomised analysis), and any statistical methods to handle missing data (eg, multiple imputation)	13
**Methods: Monitoring**			
Data monitoring	21a	Composition of data monitoring committee (DMC); summary of its role and reporting structure; statement of whether it is independent from the sponsor and competing interests; and reference to where further details about its charter can be found, if not in the protocol. Alternatively, an explanation of why a DMC is not needed	14
	21b	Description of any interim analyses and stopping guidelines, including who will have access to these interim results and make the final decision to terminate the trial	14
Harms	22	Plans for collecting, assessing, reporting, and managing solicited and spontaneously reported adverse events and other unintended effects of trial interventions or trial conduct	14
Auditing	23	Frequency and procedures for auditing trial conduct, if any, and whether the process will be independent from investigators and the sponsor	14
**Ethics and dissemination**			
Research ethics approval	24	Plans for seeking research ethics committee/institutional review board (REC/IRB) approval	15
Protocol amendments	25	Plans for communicating important protocol modifications (eg, changes to eligibility criteria, outcomes, analyses) to relevant parties (eg, investigators, REC/IRBs, trial participants, trial registries, journals, regulators)	15

For peer review only - http://bmjopen.bmj.com/site/about/guidelines.xhtml

Consent or assent	26a	Who will obtain informed consent or assent from potential trial participants or authorised surrogates, and how (see Item 32)	7
	26b	Additional consent provisions for collection and use of participant data and biological specimens in ancillary studies, if applicable	n/a
Confidentiality	27	How personal information about potential and enrolled participants will be collected, shared, and maintained in order to protect confidentiality before, during, and after the trial	11-12
Declaration of interests	28	Financial and other competing interests for principal investigators for the overall trial and each study site	18
Access to data	29	Statement of who will have access to the final trial dataset, and disclosure of contractual agreements that limit such access for investigators	15
Ancillary and post-trial care	30	Provisions, if any, for ancillary and post-trial care, and for compensation to those who suffer harm from trial participation	n/a
Dissemination policy	31a	Plans for investigators and sponsor to communicate trial results to participants, healthcare professionals, the public, and other relevant groups (eg, via publication, reporting in results databases, or other data sharing arrangements), including any publication restrictions	15
	31b	Authorship eligibility guidelines and any intended use of professional writers	15
	31c	Plans, if any, for granting public access to the full protocol, participant-level dataset, and statistical code	n/a
**Appendices**			
Informed consent materials	32	Model consent form and other related documentation given to participants and authorised surrogates	Supplementary Files 1&2
Biological specimens	33	Plans for collection, laboratory evaluation, and storage of biological specimens for genetic or molecular analysis in the current trial and for future use in ancillary studies, if applicable	11

*It is strongly recommended that this checklist be read in conjunction with the SPIRIT 2013 Explanation & Elaboration for important clarification on the items. Amendments to the protocol should be tracked and dated. The SPIRIT checklist is copyrighted by the SPIRIT Group under the Creative Commons "Attribution-NonCommercial-NoDerivs 3.0 Unported" license.

For peer review only - http://bmjopen.bmj.com/site/about/guidelines.xhtml

BMJ Open

safetxt: a safer sex intervention delivered by mobile phone messaging on sexually transmitted infections (STI) among young people in the UK- protocol for a randomised controlled trial

Journal:	*BMJ Open*
Manuscript ID	bmjopen-2019-031635.R2
Article Type:	Protocol
Date Submitted by the Author:	08-Jan-2020

Complete List of Authors:	Free, Caroline; London School of Hygiene and Tropical Medicine, Population Health<br>McCarthy, Ona; London School of Hygiene and Tropical Medicine, Population Health<br>Palmer, Melissa; London School of Hygiene and Tropical Medicine, Population Health<br>Knight, Rosemary; London School of Hygiene and Tropical Medicine, Clinical Trials Unit, MSD<br>Edwards, Phil; London School of Hygiene and Tropical Medicine, Population Health<br>French, Rebecca ; London School of Hygiene and Tropical Medicine, Social and Environmental Health Research<br>Baraitser, Paula; King's College London, Centre for Global Health<br>Hickson, Ford; London School of Hygiene and Tropical Medicine, Sigma Research<br>Wellings, Kaye; London School of Hygiene and Tropical Medicine, Social and Environmental Health Research<br>Roberts, Ian; London School of Hygiene and Tropical Medicine, Population Health<br>Bailey, Julia; University College London, Primary Care and Population Health<br>Hart, Graham; University College London, Department of Infection and Population Health<br>Michie, Susan; University College London, Centre for Outcomes Research and Effectivenes<br>Clayton, Tim; London School of Hygiene and Tropical Medicine<br>Ploubidis, George; University College London Institute of Education<br>Carpenter, James; London School of Hygiene and Tropical Medicine<br>Turner, Katy; University of Bristol<br>Devries, Karen; London School of Hygiene and Tropical Medicine<br>Potter, Kimberley; London School of Hygiene and Tropical Medicine

**Primary Subject Heading**:	Sexual health
Secondary Subject Heading:	Communication, General practice / Family practice
Keywords:	sexually transmitted infection, sexual behaviour, text message, mHealth,

	randomised controlled trial, young people

SCHOLARONE™
Manuscripts

I, the Submitting Author has the right to grant and does grant on behalf of all authors of the Work (as defined in the below author licence), an exclusive licence and/or a non-exclusive licence for contributions from authors who are: i) UK Crown employees; ii) where BMJ has agreed a CC-BY licence shall apply, and/or iii) in accordance with the terms applicable for US Federal Government officers or employees acting as part of their official duties; on a worldwide, perpetual, irrevocable, royalty-free basis to BMJ Publishing Group Ltd ("BMJ") its licensees and where the relevant Journal is co-owned by BMJ to the co-owners of the Journal, to publish the Work in this journal and any other BMJ products and to exploit all rights, as set out in our licence.

The Submitting Author accepts and understands that any supply made under these terms is made by BMJ to the Submitting Author unless you are acting as an employee on behalf of your employer or a postgraduate student of an affiliated institution which is paying any applicable article publishing charge ("APC") for Open Access articles. Where the Submitting Author wishes to make the Work available on an Open Access basis (and intends to pay the relevant APC), the terms of reuse of such Open Access shall be governed by a Creative Commons licence – details of these licences and which Creative Commons licence will apply to this Work are set out in our licence referred to above.

Other than as permitted in any relevant BMJ Author's Self Archiving Policies, I confirm this Work has not been accepted for publication elsewhere, is not being considered for publication elsewhere and does not duplicate material already published. I confirm all authors consent to publication of this Work and authorise the granting of this licence.

For peer review only

Title Page

safetxt: a safer sex intervention delivered by mobile phone messaging on sexually transmitted infections (STI) among young people in the UK- protocol for a randomised controlled trial.

Corresponding author: Caroline Free. caroline.free@lshtm.ac.uk

Authors

Caroline Free, Department of Population Health, Faculty of Epidemiology and Population Health, London School of Hygiene & Tropical Medicine, Keppel Street, London, WC1E 7HT. caroline.free@lshtm.ac.uk

Ona L McCarthy, Department of Population Health, Faculty of Epidemiology and Population Health, London School of Hygiene & Tropical Medicine, Keppel Street, London, WC1E 7HT. ona.mccarthy@lshtm.ac.uk

Melissa Palmer, Department of Population Health, Faculty of Epidemiology and Population Health, London School of Hygiene & Tropical Medicine, Keppel Street, London, WC1E 7HT. melissa.palmer@lshtm.ac.uk

Rosemary Knight, Clinical Trials Unit. Department of Medical Statistics, Faculty of Epidemiology and Population Health, London School of Hygiene & Tropical Medicine, Keppel Street, London, WC1E 7HT. rosemary.knight@lshtm.ac.uk

Phil Edwards, Department of Population Health, Faculty of Epidemiology and Population Health, London School of Hygiene & Tropical Medicine, Keppel Street, London, WC1E 7HT. phil.edwards@lshtm.ac.uk

Rebecca French, Department of Public Health Environments and Society, Faculty of Public Health and Policy, London School of Hygiene & Tropical Medicine, 15-17 Tavistock Place, London, WC1H 9SH. rebecca.french@lshtm.ac.uk

Paul Baraitser, King's Centre for Global Health, King's College London, Camberwell Sexual Health Centre, 100 Denmark Hill, London, SE5 9RS. paula_baraitser@mac.com

Ford Hickson, Department of Social and Environmental Health Research, Faculty of Public Health and Policy, London School of Hygiene & Tropical Medicine, 15-17 Tavistock Place, London, WC1H 9SH. ford.hickson@lshtm.ac.uk

For peer review only - http://bmjopen.bmj.com/site/about/guidelines.xhtml

Kaye Wellings, Department of Public health Environments and Society, Faculty of Public Health and Policy, London School of Hygiene & Tropical Medicine, 15-17 Tavistock Place, London, WC1H 9SH. kaye.wellings@lshtm.ac.uk

Ian Roberts, Department of Population Health, Faculty of Epidemiology and Population Health, London School of Hygiene & Tropical Medicine, Keppel Street, London, WC1E 7HT. ian.roberts@lshtm.ac.uk

Julia V Bailey, Department of Primary Care and Population Health, University College London, Royal Free Hospital, Rowland Hill Street, London NW3 2PF. julia.bailey@ucl.ac.uk

Graham Hart, Faculty of Population Health Sciences, University College London, Gower Street, London, WC1E 6BT. g.hart@ucl.ac.uk

Susan Michie, Department of Clinical, Educational and Health Psychology, University College London, Gower Street, London, WC1E 6BT. s.michie@ucl.ac.uk

Tim Clayton. Department of Medical Statistics. Faculty of Epidemiology and Population Health, London School of Hygiene & Tropical Medicine, Keppel Street, London, WC1E 7HT. tim.clayton@lshtm.ac.uk

George Ploubidis, Department of Social Science, Institute of Education, University College London, Gower Street, London, WC1E 6BT. g.ploubidis@ucl.ac.uk

James Carpenter, Department of Medical Statistics. Faculty of Epidemiology and Population Health, London School of Hygiene & Tropical Medicine, Keppel Street, London, WC1E 7HT. james.carpenter@lshtm.ac.uk

Katy Turner, Bristol Veterinary School, University of Bristol, Churchill Building, Langford Campus, Bristol, BS40 5DU katy.turner@bristol.ac.uk

Karen Devries, Faculty of Public Health and Policy. LSHTM, 15-17 Tavistock Place, London WC1H 9SH. karen.devries@lshtm.ac.uk

Kimberley Potter, Clinical Trials Unit. Department of Medical Statistics, Faculty of Epidemiology and Population Health, London School of Hygiene & Tropical Medicine, Keppel Street, London, WC1E 7HT. kimberley.potter@lshtm.ac.uk

For peer review only - http://bmjopen.bmj.com/site/about/guidelines.xhtml

Abstract

Introduction

Young people aged 16-24 have the highest prevalence of genital chlamydia and gonorrhoea compared to other age groups and re-infection rates following treatment are high. Long-term adverse health effects include sub-fertility and ectopic pregnancy, particularly among those with repeated infections. We developed the safetxt intervention delivered by text message to reduce sexually transmitted infection (STI) by increasing partner notification, condom use and (STI) testing among young people in the United Kingdom (UK).

Methods and analysis

A single blind randomised trial to reliably establish the effect of the safetxt intervention on chlamydia and gonorrhoea infection at one year. We will recruit 6250 people aged 16-24 years who have recently been diagnosed with chlamydia, gonorrhoea or non-specific urethritis from health services in the UK. Participants will be allocated to receive the safetxt intervention (text messages designed to promote safer sexual health behaviours) or to receive the control text messages (monthly messages asking participants about changes in contact details) by an automated remote online randomisation system.

The primary outcome will be the cumulative incidence of chlamydia and gonorrhoea infection at one year assessed by nucleic acid amplification tests. Secondary outcomes include partner notification, correct treatment of infection, condom use, and STI testing prior to sex with new partners.

Ethics and dissemination

Ethics approval was obtained from NHS Health Research Authority - London – Riverside Research Ethics Committee (REC reference: 15/LO/1665) and the LSHTM. We will submit the results of the trial for publication in peer reviewed journals.

Trial registration

International Standard Randomised Controlled Trials Number: ISRCTN64390461. Registered on 17th March 2016. *World Health Organization trial registration dataset*

Available at: http://apps.who.int/trialsearch/Trial2.aspx?TrialID=ISRCTN64390461

Trial Protocol version: 12 19th July 2018

For peer review only - http://bmjopen.bmj.com/site/about/guidelines.xhtml

Strengths and limitations of this study

- The study design is a large randomised trial to assess the effect of 'safetxt', a safer sex intervention delivered by text message, on objectively measured STI (chlamydia and gonorrhoea) infection at one year.
- The use of an independent remote computer-based randomisation system linked to an automated message delivery system ensures allocation concealment.
- The secondary outcomes will assess the effect of the intervention on safer sex behaviours: partner notification, condom use and testing before sex with a new partner.
- The process evaluation will explore which intervention components are effective and has the potential to generate general principles to inform similar interventions in the future.
- The inability to blind participants receiving a behavioural intervention is a limitation of the trial, which could introduce bias in the ascertainment of self-reported outcomes.

Keywords

Sexually transmitted infection (STI); sexual behaviour; young people; mHealth; randomised controlled trial, text message.

For peer review only - http://bmjopen.bmj.com/site/about/guidelines.xhtml

INTRODUCTION

Younger people aged 16-24 bear the heaviest burden of sexually transmitted infections (STIs) such as chlamydia and gonorrhoea, and their long-term adverse health effects including ectopic pregnancy and subfertility (1, 2). The risk of adverse health effects increases with repeated infections, and re-infection rates following treatment are high: up to 30% for chlamydia and 12% for gonorrhoea at one year (3). Those with an STI are more likely to acquire further STIs and HIV, if exposed. There are marked inequalities in sexual health; STIs are positively associated with lower educational level (4) and living in more deprived areas (1, 5, 6).

Partner notification, condom use and STI testing can reduce infection and re-infection. There is some evidence that existing interventions delivered face-to-face that target condom use and/or STI testing may be effective, but they are limited in their reach or too costly for widespread application (7). Existing interventions delivered via the media have high reach but their effects have yet to be established (8). Effective ways to increase partner notification in specialist and primary care settings are needed (9, 10).

Mobile phones have the potential to provide effective, low cost health behaviour support (11, 12). However, evidence for the effect of mobile phone support for safer sex behaviours such as condom use, partner notification and STI testing is equivocal (13-15). Interventions have targeted testing for STI (16-21), delaying resumption of sexual activity until 42 days after circumcision (22) and condom use (17, 18, 23, 24), but none have aimed to increase partner notification. The effect of mobile phone based interventions on STI is not known.

In the UK, 99% of 16-24 year olds are mobile phone users (ONS, 2012) and mobile phone ownership is high across all socio-economic groups. In research leading to this trial, it was demonstrated that interactive support via text message is particularly acceptable in the area of sexual health intervention (15, 25, 26).

The safetxt trial builds on the successful intervention development work and pilot trial (15, 26, 27). To develop the intervention, we convened a working group including experts in health psychology, contraception research, contraception service provision, IT and the development of interventions delivered by mobile phone. The intervention messages were developed based on: behaviour change theory; evidence-based behaviour change techniques; the content of effective face-to-face safer sex interventions; the factors known to influence safer sex behaviours; the views of 82 young people collected in focus groups and a questionnaire completed by 100 people aged 16-24 (15). The theory and evidence-based intervention is designed to reduce STIs in young

For peer review only - http://bmjopen.bmj.com/site/about/guidelines.xhtml

people by supporting them in telling a partner about an infection, using condoms and obtaining testing before unprotected sex with a new partner. In a qualitative study with young people, recipients reported that the tone, language, content, and frequency of messages was appropriate (26). Messages reportedly increased knowledge and confidence in how to use condoms and reduced stigma enabling them to tell a partner about an STI. Sharing messages with their partner enabled participants to negotiate condom use.

The pilot trial demonstrated that methods of recruitment, randomisation, intervention delivery and follow-up were successful and that a full-scale randomised controlled trial of the safetxt intervention is feasible (27).

Objectives

The primary objective of this trial is to quantify the effects of the safetxt intervention compared to a control group receiving usual care and messages about trial participation on chlamydia or gonorrhoea infection at one year. Secondary objectives are to determine the effects of safetxt on partner notification and condom use at 4 weeks and on condom use and STI testing at one year. Which intervention components are effective will be explored by collecting data on the theoretical constructs on the pathway to behaviour change influenced by the intervention components. The cost-effectiveness of the intervention will be established.

METHODS AND ANALYSIS

Design and setting

Safetxt is a single blind parallel group randomised superiority trial with a 1:1 allocation ratio designed to establish the effects of a safer sex intervention delivered by text message on the cumulative incidence of chlamydia and gonorrhoea infection at one year. (See Figure 1 for the trial flow chart).

Potential participants testing positive for chlamydia, gonorrhoea or diagnosed with non-specific urethritis (NSU) will be identified from UK STI testing services by research staff based at the service (a full list of participating services is available at http://safetxt.lshtm.ac.uk/participating-sites/). The intervention is delivered by text message in the community. Trial recruitment started on the 1st April 2016. Completion of follow up and closure of the data set is planned for early spring 2020.

Characteristics of participants

For peer review only - http://bmjopen.bmj.com/site/about/guidelines.xhtml

Inclusion criteria

Participants will be eligible if they are between 16 and 24 years of age, own a personal mobile phone, are able to provide informed consent and who, in the 2 weeks prior to their recruitment, have either been diagnosed with chlamydia, gonorrhoea, or NSU, or have started treatment for chlamydia, gonorrhoea or NSU.

Exclusion criteria

Participants will be excluded if they are known to be a sexual partner of someone already recruited to the trial.

Recruitment and consent

Research staff based at STI testing services will identify potential participants. They will provide potential participants with trial information at one of three time points, i.e. when potential participants (i) attend the service and are diagnosed with chlamydia, gonorrhoea or NSU; (ii) receive positive test results for chlamydia or gonorrhoea by phone; or (iii) collect treatment for chlamydia, gonorrhoea or NSU from services.

Research staff will provide potential participants with verbal and written information (Supplementary file 1) about the trial and/or participants will view trial information on the website. Participants will be able to join the trial by either providing informed written consent to the research staff, or by providing consent online (Supplementary file 2) at the trial website or site staff can ask eligible participants if they are happy for their details to be given to research assistants based at the London School of Hygiene & Tropical Medicine Clinical Trials Unit (LSHTM CTU). In this case, LSHTM CTU will contact participants to recruit to the trial. Strategies for achieving adequate enrolment include site visits, training, feedback and rewards, sharing successful recruiting strategies and newsletters.

Allocation and blinding

Participants will be randomly allocated (1:1 ratio), using a computer-based randomisation system, to a safer sex intervention delivered by text messaging, or to a control group, ensuring allocation concealment. Allocation concealment is ensured as the randomisation system is independent, automated and remote.

An electronic link to the computer-based randomisation programme will result in the generation of a research number and allocation to the intervention or control group. The system will then automatically deliver intervention or control group texts according to the allocation.

For peer review only - http://bmjopen.bmj.com/site/about/guidelines.xhtml

Given the nature of the intervention, participants will be aware of their assigned group. Research assistants entering data, laboratory staff and researchers conducting the final analysis are masked to allocation until after the analysis is complete.

Interventions:

Safetxt Intervention group

The intervention aims to increase safer sex in three ways: i) encouraging participants to correctly follow STI treatment instructions including informing partner(s) about infection; ii) promoting condom use with new or casual partners; and iii) encouraging participants to obtain testing for STI prior to unprotected sex. Participants in the intervention group will receive regular messages delivered by text message in community settings according to a predetermined schedule. The intervention was informed by the capability, opportunity and motivation model of behaviour (COM-B)(28). It aims to influence the knowledge, beliefs, self-efficacy, skills, social and interpersonal influences that have important effects on motivation, capability and opportunity to reduce sexual risk behaviour.

Over the first ten days participants are sent messages targeting engagement with the intervention, taking treatment, avoiding sex for 7 days after treatment and telling partner (s) about an infection. These messages provide non-judgemental, non-stigmatising information about STI. They provide suggestions about when, where, and how to tell partner(s) about an infection and examples of how others told partners covering a range of different types of relationship (e.g. casual, long term).

Messages then target condom use and testing for STIs before having sex without a condom with a new partner. Topics cover risk assessment, instructions on how to use condoms, positive aspects of condom use, tips on preventing condom problems and examples of how others resolved condom use problems. Participants are prompted to think about their own success in achieving safer sex strategies, risks they had taken and what they could do differently in the future. Messages included advice regarding testing before unprotected sex with a new partner. Participants are sent links to: services; support for those concerned about partner violence; and web-based information regarding contraception, alcohol and sexual risk, how to use a condom and general communication about sex. The messages provide social support for safer sex behaviours and acknowledge participants experiences.

The intervention employs educational, enabling and incentivising behaviour change functions and twelve behaviour change techniques: information about health consequences of behaviour;

For peer review only - http://bmjopen.bmj.com/site/about/guidelines.xhtml

instruction on how to carry out the behaviour; demonstrations of risk reduction behaviour; social support; emotional support; social rewards; non-specific incentives; encouragement to add objects to the environment; anticipated regret; problem solving; action planning techniques; and reframing (29) . The information on safer sexual practices is in accordance with existing guidelines.

The messages are tailored according to gender and sexual orientation. Women who have sex with men only (WSM), men who have sex with men only (MSM), men who have sex with men and women (MSMW), women who have sex with men and women (WSMW) are sent messages about how others had negotiated condom use. WSM and men who have sex with women (MSW) are sent messages about emergency contraception. MSM and MSMW are sent messages about post exposure prophylaxis (PEP). Women who have sex with women only (WSW) are not sent messages about condom use. The information provided is specific to the STI diagnosed. This tailoring results in different numbers of messages being sent to those of different gender and sexual orientation.

The core message sets include: 74 messages for WSM and WSMW; 42 messages for WSW; 69 messages for MSW and 79 messages for MSMW; and 76 messages for MSM. Recipients can request additional messages on specific topics. Participants are sent: 4 messages per day for days 1-3, then 1-2 messages per day for days 4-28, then 2-3 messages per week for month 2, and 2-5 messages per month for months 3-12.

Control group

Participants in the control group will receive a monthly untailored text message asking for information about changes in postal or email addresses. Example control group message: 'Thank you for taking part in the texting study. Remember to let us know if your contact details have changed by replying to this text or emailing safetxt@lshtm.ac.uk'

All participants will receive usual care and will be free to seek any other existing service or support they wish. The control group is not attention matched as during the pilot work young people reported that it was irritating to receive the same number of messages as the intervention group about another health topic, when they had just been diagnosed with an STI. If the trial shows a benefit it is possible that this could be due to simply receiving the number of messages involved in the intervention, rather than the message content. However, given the lack of effect of some mobile phone message based interventions in sexual health this seems unlikely (14)

For peer review only - http://bmjopen.bmj.com/site/about/guidelines.xhtml

All messages are sent automatically from a large database to an aggregator, which conveys messages to each participant in the community via their network. The success of delivery of messages at each step is monitored by the aggregator and computer system that generates the messages. A member of the trial team will automatically be notified if there is any failure in the delivery of messages. All participants will be able to set embargoed times when they do not want to receive messages. Participants will be able to stop text messages, but continue with the trial follow up, by texting 'stop' to the short code number.

Outcomes

Our primary outcome will be the cumulative incidence of chlamydia and gonorrhoea infection at one year assessed by nucleic acid amplification tests: urine for men (with additional pharyngeal and anal swabs for MSM) and self-taken vulvo-vaginal swab for women.

Secondary outcomes

Secondary outcomes at four weeks

Behaviours

- Whether participants took the (prescribed antibiotic) treatment and avoided sex for 7 days after treatment
- Whether they told the last person they had sex with before testing positive, that they needed to get treatment
- Clinic attendance by partner for treatment (identified from clinic records)
- Condom use at last sex

Process outcomes – scores of the theoretical constructs underlying the components of the intervention (behaviour change mediators):

- Attitudes towards partner notification
- Self–efficacy in telling a partner about an infection
- Self–efficacy in negotiating condom use
- Correct condom use self-efficacy
- Knowledge related to STIs

For peer review only - http://bmjopen.bmj.com/site/about/guidelines.xhtml

Secondary outcomes at one year

STIs

- Diagnosed with any STI after joining the trial according to self-report confirmed by postal test results and clinic records

Behaviours

- Condom use at last sex
- Number of sexual partners since joining the trial
- Sex with someone new since joining the trial
- Condom use at first sex with most recent new partner
- Self-reported sexually transmitted infection testing for self - prior to first sex with most recent new partner
- Sexually transmitted infection testing for self - prior to first sex with most recent new partner (confirmed according to clinic record that testing occurred)
- Participant's report as to whether their most recent new partner was tested for sexually transmitted infection prior to sex with them
- Number of sexual partners since joining the trial
- Reading and sharing of intervention content
- Number of text messages read
- Whether anyone else read the messages, if yes how did the participant feel about them reading the messages?
- Reading someone else's messages in the trial (to be reported for control group participants)
- Someone else in the trial reading participants' messages (to be reported for intervention group participants)

Potential harms

- Car accident where the participant was the driver in the past year (30)
- Experience of partner violence in the past year (31)

Data collection

The primary outcome will be assessed using chlamydia and gonorrhoea tests collected by post at one year and clinic records of completed tests. STI test kits for chlamydia and gonorrhoea will be posted (in P650 standard packaging) to respondents. Directions in the pack will ask participants

For peer review only - http://bmjopen.bmj.com/site/about/guidelines.xhtml

to provide a vaginal swab (women), a urine sample (men), and additional anal and pharyngeal samples (men who have sex with men) and post it in the prepaid and addressed envelope to the laboratory. Test kits will be identified by lab number only, rendering the laboratory staff blind to the participant's allocation. The accredited laboratory will destroy samples after testing is completed.

Results of the STI testing will be reported on the secure trial lab site by lab code only. Clinic records will be checked to identify participants who have had a positive STI diagnosis since joining the study, confirm self-reported STI testing and partner attendance for treatment.

At 4 weeks and at one-year, postal questionnaires will be sent to all participants to collected self-reported data. Non-responders will be contacted by any method the participant agrees to at enrolment (post, email, text message, telephone call). Participants can directly enter self-reported outcome data via a web-based data entry form. Paper based self-reported outcome data or self-reported data collected by telephone will be directly entered into the web-based data entry form by a trial assistant blinded to treatment allocation.

Participants will be sent a £5 unconditional incentive with each postal request i.e. when sending the 4 weeks questionnaire and 1-year test and questionnaire. Participants who return the test sample will be sent £20. See Figure 2 for the Schedule of enrolment, interventions and assessments. All outcome data will be collected on participants who choose to stop messages unless they withdraw from the trial.

Data management

Anonymised research data will be held on a secure system and password protected. All data systems will be set up with checks to alert the trial assistants if data being entered are illogical, inconsistent or incomplete.

Personal details will be stored on a password protected computer held on a secure server at the LSHTM. In accordance with the Data Protection Act of 1998 (32), this information will be stored separately from any anonymised research data, and will be deleted at the end of the study.

Any paper-based data will be locked in a cabinet within a room. All trial procedures are in accordance with the principles of Good Clinical Practice. Essential documents of the sponsor/trial organisers and investigators will be retained for at least ten years after completion of the trial.

Sample size

For peer review only - http://bmjopen.bmj.com/site/about/guidelines.xhtml

The study is powered for the primary outcome measure of cumulative incidence of chlamydia and/or gonorrhoea infection at one year. Two main factors determine the number of participants needed for this trial: the estimated event rate, and the size of the treatment effect. Our estimates are based on the following data:

Estimated event rate

The estimated event rate for the cumulative incidence of STI at 1 year is 20%, based on the event rate in cohort studies and the pilot trial (3).

Size of treatment effect

Because the intervention can be administered to large populations at low cost, even a modest reduction in treatable STIs would be worthwhile. The trial has therefore been designed to detect a reduction in chlamydia or gonorrhoea infection of 20% versus 16% (RR 0.8), which is similar to the effects of face-to-face safer sex interventions (7).

Sample size calculation

In the pilot trial 2% of the control group viewed messages delivered to intervention participants. If the real difference in STI infection at one year follow up is 20% versus 16% then with contamination of 2% the trial would detect a difference of 19.9% vs 16%. (Calculated based on 2% of the control group having an infection rate the same as the intervention group = (98% x 20%) + (2% x16%) = 19.9%).

Five thousand participants would provide 90% power to detect this difference, allowing for 20% losses to follow-up.

The TSC reviewed the blinded event rate after 546 patients had completed 12 months follow up and recommended an increase in the sample size to 6250 due to a lower than expected event rate of 15.6%.

Analysis

A detailed statistical analysis plan (SAP) will be finalised and submitted to the TSC before the end of the trial and unblinding. For the primary outcome and other binary outcomes, we will estimate any differences between the groups using logistic regression and we will report odds ratios with 95% confidence intervals and p-values, adjusting for key baseline predictors of outcome. These will specified in advance in the SAP. For continuous outcomes, we will use linear regression to test for a difference in mean scores between the arms. All analyses will be based on the intention-to-treat principle.

For peer review only - http://bmjopen.bmj.com/site/about/guidelines.xhtml

For the primary analysis Multiple Imputation by Chained Equations (MICE) will be used in order to account for missing data (33). MICE makes appropriate assumptions for accommodating missing data in the analysis based on the predictors of outcome and the predictors of loss to follow up. MICE is recognised as a way of reducing bias and increasing precision of trial results and is increasingly used as the primary analysis in randomised controlled trials (12, 33). One hundred imputed datasets will be generated and the point estimates and standard errors will be combined using Rubin's rules. We will perform information-anchored sensitivity analysis using the delta-method (34).

Supplementary analyses.

Before fitting our primary analysis model, we need to identify appropriate auxiliary variables to include in the imputation model. Such auxiliary variables should have information about the missing outcome. Specifically, an independent statistician will use a dataset without the treatment variable and perform regression analyses to identify key predictors of outcomes and which combination of baseline variables and time to negative STI test in clinic should be included as auxiliary variables in the imputation model. We will supplement this by performing and reporting a complete case analysis (where any participants with missing information on any covariate or outcome shall be excluded).

Sub group analyses

A limited number of subgroup analyses will be undertaken adjusting for other key predictors of outcome. Subgroup analyses include: age (16-19, 20-24), sexual orientation (men who have sex with men or men and women, men who have sex only with women, women who have sex with men or men and women, women who only have sex with women), ethnic group (Caucasian, Black, other) and age at which left education (16 or under, 17 or over). The subgroups will be analysed by inclusion of an interaction term between treatment group and the subgroup in the appropriate regression model for the outcome. Data will be presented by categories of subgroup using effect estimates and 99% confidence intervals. For each subgroup analysis, missing outcomes will be imputed consistent with the hypothesis of an interaction (35) .

Process outcomes

Candidate questionnaire items were identified for the measurement of the following constructs: attitudes towards partner notification; self–efficacy in telling a partner about an infection; self–efficacy in negotiating condom use; correct condom use self-efficacy; knowledge related to STIs. At four weeks, each of the five process outcomes were measured using the candidate questionnaire items (3 to 5 items per process outcome). Based on these items, we will carry out confirmatory factor analysis (CFA) to refine and identify a valid measurement model. For the

For peer review only - http://bmjopen.bmj.com/site/about/guidelines.xhtml

For peer review only

main trial analyses, the final measurement model will be combined with the intervention group allocation, along with key baseline predictors, extending the measurement model to a Multiple Causes Multiple Indicators (MIMIC) model. This model will be used to estimate the effects of the intervention on the five hypothesised process outcomes.

Economic evaluation

The economic modelling required to assess the cost-effectiveness of the intervention will estimate the annual probability of members of the target group acquiring chlamydia, gonorrhoea and NSU with and without the intervention (based on the experience of those in the control and intervention arms of the trial). Detailed information will be collected on the costs of delivering the intervention. Secondary sources will be used to estimate the future NHS costs avoided as a consequence of avoided infections.

Patient involvement

Patients were involved in the development of the intervention (15) and the design of the trial materials and follow-up procedures (36). A patient of also a member of the Trial Steering Committee. Young people will be involved in the dissemination of the trial results.

Monitoring

The safetxt trial will not have a separate Data Monitoring Committee (DMC). No interim analyses of intervention effects will be conducted since the trial is of a behavioural support intervention unlikely to cause harm, and therefore, there are no stopping rules. Analysis of intervention effects will be conducted once at the end of the trial. The sponsor may audit the trial per their own risk assessment and schedule.

Harms will be assessed by self-reported data. Car accidents are the only demonstrated harm resulting from text messaging, hence the intention to collect data regarding involvement in road traffic accidents. The safetxt intervention aims to increase partner notification of STI status. Fear of partner violence has been reported to be a barrier to partner notification and partner notification has been identified as a factor which may trigger partner violence. However, no randomised controlled trials targeting increased partner notification detected a difference in partner violence between the control intervention groups. Data will be collected regarding the experience of partner violence at 12 months.

In addition, participants will be asked an open-ended question in the 12 month questionnaire: "Did anything good or bad happen as a result of being involved in the study or receiving the text messages? Please describe." The Clinical Trials Unit will keep detailed records of all adverse

For peer review only - http://bmjopen.bmj.com/site/about/guidelines.xhtml

events reported. Reports will be reviewed by the Principal Investigator and the TSC to consider intensity, causality and expectedness. As appropriate, these will be reported to the sponsor and the Ethics Committee.

Ethics and Dissemination

Ethical approval for this trial was provided by the NHS Health Research Authority - London – Riverside Research Ethics Committee (REC reference: 15/LO/1665) and the LSHTM. All trial participants will provide written informed consent prior to enrolment in the trial. Participants' can withdraw from the trial on request. Participant will receive usual NHS care there is no ancillary care.

Any modifications to the protocol will be approved by the ethics committee prior to implementation. Records of any important modifications will be submitted as an addendum the journal in which this protocol is published.

Trial results will be published open-access in peer-review journals. Authorship will be on the basis of meeting the criteria recommended by the International Committee of Medical Journal Editors (ICMJE).

After the publication of our main trial findings, the anonymised trial dataset will be available on request from the corresponding author.

DISCUSSION

STIs such as chlamydia and gonorrhoea confer a heavy burden of disease among young people with long-term sequelae such as infertility. Safer sex behaviours such as condom use, notifying partner(s) about an existing STI and STI testing reduce the risk of STI, but young people may lack the knowledge, confidence and skills needed to adopt these behaviours. Safer sex interventions delivered by mobile phone are acceptable to young people and show promise in increasing safer sex behaviours, but to date their effects on STI are not reliably known.

If it proves to be effective, this low-cost intervention could have an important impact in improving sexual health and reducing inequalities among young people in the UK. There is likely to be international interest in the impact of the intervention as short written messages delivered via mobile phones are increasingly used for behavioural support worldwide and sexually transmitted infections remain an important cause of morbidity and mortality.

For peer review only - http://bmjopen.bmj.com/site/about/guidelines.xhtml

Supplementary files

Supplementary File 1. Patient Information sheet

Supplementary File 2. Patient consent form

List of abbreviations

CTU – Clinical Trials Unit

GCP – Good Clinical Practice STI - Sexually transmitted infection

HIV – Human Immunodeficiency Virus

LSHTM - London School of Hygiene & Tropical Medicine

MICE – Multiple Imputation by Chained Equations

MSM – Men who have sex with men

NAAT - Nucleic Acid Amplification Test

NSU – Non-Specific Urethritis

REFERENCES

1. Sonnenberg P, Clifton S, Beddows S, Field N, Soldan K, Tanton C, et al. Prevalence, risk factors, and uptake of interventions for sexually transmitted infections in Britain: findings from the National Surveys of Sexual Attitudes and Lifestyles (Natsal). Lancet. 2013;382(9907):1795-806.
2. Cates W, Jr., Wasserheit JN. Genital chlamydial infections: epidemiology and reproductive sequelae. Am J Obstet Gynecol. 1991;164(6 Pt 2):1771-81.
3. Hosenfeld CB, Workowski KA, Berman S, Zaidi A, Dyson J, Mosure D, et al. Repeat infection with Chlamydia and gonorrhea among females: a systematic review of the literature. Sex Transm Dis. 2009;36(8):478-89.
4. Sheringham J, Mann S, Simms I, Stafford M, Hart GJ, Raine R. It matters what you measure: a systematic literature review examining whether young people in poorer socioeconomic circumstances are more at risk of chlamydia. Sexually Transmitted Infections. 2013;89(2):175-80.
5. De Waroux OLP, Harris R, Hughes G, Crook P. The epidemiology of gonorrhoea in London: a Bayesian spatial modelling approach. Epidemiology and infection. 2014;142(01):211-20.
6. Hughes G, Nichols T, Peters L, Bell G, Leong G, Kinghorn G. Repeat infection with gonorrhoea in Sheffield, UK: predictable and preventable? Sexually Transmitted Infections. 2012.
7. Free C, Roberts IG, Abramsky T, Fitzgerald M, Wensley F. A systematic review of randomised controlled trials of interventions promoting effective condom use. J Epidemiol Community Health. 2011;65(2):100-10.

For peer review only

8. French RS, Bonell C, Wellings K, Weatherburn P. An exploratory review of HIV prevention mass media campaigns targeting men who have sex with men. BMC Public Health. 2014;14:616.
9. Estcourt C SL, Mercer CH, Copas A, Muniia P, Rait G, et al. Can accelerated partner therapy (APT) improve outcomes of partner notification for women diagnosed with genital chlamydia in primary care settings: A pilot randomized controlled trial in general practice and community sexual health services. Sexually Transmitted Diseases Conference; Conference start: 20140609 Conference end: 20140612; Atlanta, GA United States2014. p. S32-S3.
10. Control ECfDPa. Public health benefits of partner notification for sexually transmitted infections and HIV. Stockholm: European Centre for Disease Prevention and Control: 2013.
11. Guerriero C, Cairns J, Roberts I, Rodgers A, Whittaker R, Free C. The cost-effectiveness of smoking cessation support delivered by mobile phone text messaging: Txt2stop. Eur J Health Econ. 2013;14(5):789-97.
12. Free C, Knight R, Robertson S, Whittaker R, Edwards P, Zhou W, et al. Smoking cessation support delivered via mobile phone text messaging (txt2stop): a single-blind, randomised trial. Lancet. 2011;378(9785):49-55.
13. Free C, Phillips G, Galli L, Watson L, Felix L, Edwards P, et al. The effectiveness of mobile-health technology-based health behaviour change or disease management interventions for health care consumers: a systematic review. PLoS Med. 2013;10(1):e1001362.
14. Burns K, Keating P, Free C. A Systematic Review of mHealth RCTs for Sexual Health. BMC Public Health. 2016;16:778.
15. Free C, McCarthy O, French R, Wellings K, Michie S, Roberts I, et al. Can text messages increase safer sex behaviours in young people? Intervention development and pilot randomised controlled trial. Health Technol Assess 2016;20(57).
16. de Tolly K, Skinner D, Nembaware V, Benjamin P. Investigation into the use of short message services to expand uptake of human immunodeficiency virus testing, and whether content and dosage have impact. Telemed J E Health. 2012;18(1):18-23.
17. Gold J, Lim MS, Hellard ME, Hocking JS, Keogh L. What's in a message? Delivering sexual health promotion to young people in Australia via text messaging. BMC Public Health. 2010;10:792.
18. Lim MS, Hocking JS, Aitken CK, Fairley CK, Jordan L, Lewis JA, et al. Impact of text and email messaging on the sexual health of young people: a randomised controlled trial. J Epidemiol Community Health. 2012;66(1):69-74.
19. Field N, Mercer CH, Sonnenberg P, Tanton C, Clifton S, Mitchell KR, et al. Associations between health and sexual lifestyles in Britain: findings from the third National Survey of Sexual Attitudes and Lifestyles (Natsal-3). Lancet. 2013;382(9907):1830-44.
20. Downing SG, Cashman C, McNamee H, Penney D, Russell DB, Hellard ME. Increasing chlamydia test of re-infection rates using SMS reminders and incentives. Sex Transm Infect. 2013;89(1):16-9.
21. Mugo PM, Wahome EW, Gichuru EN, Mwashigadi GM, Thiong'o AN, Prins HA, et al. Effect of Text Message, Phone Call, and In-Person Appointment Reminders on Uptake of Repeat HIV Testing among Outpatients Screened for Acute HIV Infection in Kenya: A Randomized Controlled Trial. PLoS ONE [Electronic Resource]. 2016;11:e0153612.
22. Odeny TA, Bailey RC, Bukusi EA, Simoni JM, Tapia KA, Yuhas K, et al. Effect of text messaging to deter early resumption of sexual activity after male circumcision for HIV prevention: a randomized controlled trial. J Acquir Immune Defic Syndr. 2014;65(2):e50-7.
23. Suffoletto B, Akers A, McGinnis KA, Calabria J, Wiesenfeld HC, Clark DB. A sex risk reduction text-message program for young adult females discharged from the emergency department. J Adolesc Health. 2013;53(3):387-93.

For peer review only - http://bmjopen.bmj.com/site/about/guidelines.xhtml

24. Jones R, Hoover DR, Lacroix LJ. A randomized controlled trial of soap opera videos streamed to smartphones to reduce risk of sexually transmitted human immunodeficiency virus (HIV) in young urban African American women. Nurs Outlook. 2013;61(4):205-15 e3.
25. Thomas N, Murray E, Rogstad KE. Confidentiality is essential if young people are to access sexual health services. Int J STD AIDS. 2006;17(8):525-9.
26. French RS, McCarthy O, Baraitser P, Wellings K, Bailey JV, Free C. Young People's Views and Experiences of a Mobile Phone Texting Intervention to Promote Safer Sex Behavior. JMIR mHealth and uHealth. 2016;4(2).
27. McCarthy OL, French RS, Baraitser P, Roberts I, Rathod SD, Devries K, et al. Safetxt: a pilot randomised controlled trial of an intervention delivered by mobile phone to increase safer sex behaviours in young people. BMJ Open. 2016;6(12).
28. Michie S, M van Stralen M, West R. The behaviour change wheel: A new method for characterising and designing behaviour change interventions

2011;6:42.
29. Michie S, Wood C, Johnston M, Abraham C, Francis J, Hardeman W. Behaviour change techniques: the development and evaluation of a taxonomic method for reporting and describing behaviour change interventions. Health Technology Assessment. 2015;19(99):1-188.
30. Rothman K. Epidemiological evidence on health risks of cellular telephones. . Lancet. 2000;356:1837-40.
31. Decker M, Miller E, McCauley H, Tancredi D, Levenson R, Waldman J, et al.

Intimate partner violence and partner notification of sexually transmitted infections among adolescent and young adult family planning clinic patients. Int J STD AIDS. 2011;22(6):345-7.
32. Data Protection Act. 1998.
33. Sterne JA, White IR, Carlin JB, Spratt M, Royston P, Kenward MG, et al. Multiple imputation for missing data in epidemiological and clinical research: potential and pitfalls. BMJ. 2009;338:b2393.
34. Cro S, Carpenter J, Kenward MIasataa. Information anchored sensitivity analysis: theory and application. Journal of the Royal Statistical Society. 2018;182:623-45.
35. Carpenter JR, Kenward M. Multiple imputation and its application. Chichester: Wiley; 2013.
36. McCarthy O, French R, Roberts I, Free C. Simple steps to develop trial follow-up procedures. Trials. 2016;17(28).

Figures

Figure 1. Trial flow chart

Figure 2. Schedule of enrolment, interventions and assessments

Authors' contributions

CF conceived the study, contributed to the development of the intervention and statistical analysis plan, is a member of the Trial Management Group, and had overall responsibility for the design and conduct of the trial and writing the protocol.

For peer review only - http://bmjopen.bmj.com/site/about/guidelines.xhtml

OM contributed to the development of the intervention, contributed to the statistical analysis plan, is a member of the Trial Management Group, contributed to the trial design, the refinement of the study protocol and approved the manuscript.

MP contributed to the statistical analysis plan, is a member of the Trial Management Group, contributed to the process evaluation design, contributed to the trial design, the refinement of the study protocol and approved the manuscript.

RK is a member of the Trial Management Group, is are responsible for the day-to-day management of the trial, contributed to the trial design, the refinement of the study protocol and approved the manuscript.

PE is the trial statistician who provides general statistical oversight, contributed to the statistical analysis plan, contributed to the trial design, the refinement of the study protocol and approved the manuscript.

RF contributed to the development of the intervention, provided input on the trial outcome measures, contributed to the trial design, the refinement of the study protocol and approved the manuscript.

PB contributed to the development of the intervention, contributed to the trial design, the refinement of the study protocol and approved the manuscript.

FH adapted the intervention messages for men who have sex with men, contributed to the trial design, the refinement of the study protocol and approved the manuscript.

KW contributed to the development of the intervention, provided input on the trial outcome measures, contributed to the trial design, the refinement of the study protocol and approved the manuscript.

IR provided expertise on trial design, contributed to the refinement of the study protocol and approved the manuscript.

JB contributed to the development of the intervention, the trial design, the refinement of the study protocol and approved the manuscript.

GH provided fundamental intellectual input into the design of the trial, the refinement of the study protocol and approved the manuscript.

For peer review only

SM provided behavioural science expertise, which informed the development of the intervention, contributed to the trial design, the refinement of the study protocol and approved the manuscript.

TC provided statistical expertise regarding the supplementary and subgroup analyses, provided general statistical advice, contributed to the trial design, the refinement of the study protocol and approved the manuscript.

GP provided statistical expertise for the Generalised Latent Variable Modelling framework, contributed to the trial design, the refinement of the study protocol and approved the manuscript.

JC provided statistical expertise regarding multiple imputation, contributed to the trial design, the refinement of the study protocol and approved the manuscript..

KT leads the economic evaluation, contributed to the trial design, the refinement of the study protocol and approved the manuscript.

KD contributed to the development of the intervention, contributed to the trial design, the refinement of the study protocol and approved the manuscript.

KP is a member of the Trial Management Group, is are responsible for the day-to-day management of the trial, contributed to the trial design, the refinement of the study protocol and approved the manuscript.

Funding

This trial is funded by the National Institute for Health Research Public Health Research (NIHR PHR) Programme (project number 14/182/07). The funding body have no role in any aspect of the design and conduct of this study, or the decision to submit the report for publication.

Competing interests

The authors declare that they have no competing interests.

Ethics approval

Ethics approval for this trial was provided by the NHS Health Research Authority - London – Riverside Research Ethics Committee (REC reference: 15/LO/1665) and the LSHTM.

Trial Sponsor

For peer review only - http://bmjopen.bmj.com/site/about/guidelines.xhtml

London School of Hygiene & Tropical Medicine is the main research sponsor for this trial. The sponsor had no role in any aspect of the design and conduct of this study, or the decision to submit the report for publication. For further information regarding the sponsorship conditions, please contact the Research Governance and Integrity Office:

London School of Hygiene & Tropical Medicine, Keppel Street, London, WC1E 7HT

Tel: +44 207 927 2626 Email: patricia.henley@lshtm.ac.uk

Trial Steering Committee

The Trial Steering Committee (TSC) may recommend changes to the overall trial management processes for which there is evidence the current process may compromise the conduct of the trial or the safety of participants. The members of the TSC are: Professor Pippa Oakeshott (Chair), Dr Andrew Copas, Professor Michael Ussher, Mr Colum McGrady, Dr Michael Brady, and Professor Caroline Free.

Word count 3997

For peer review only

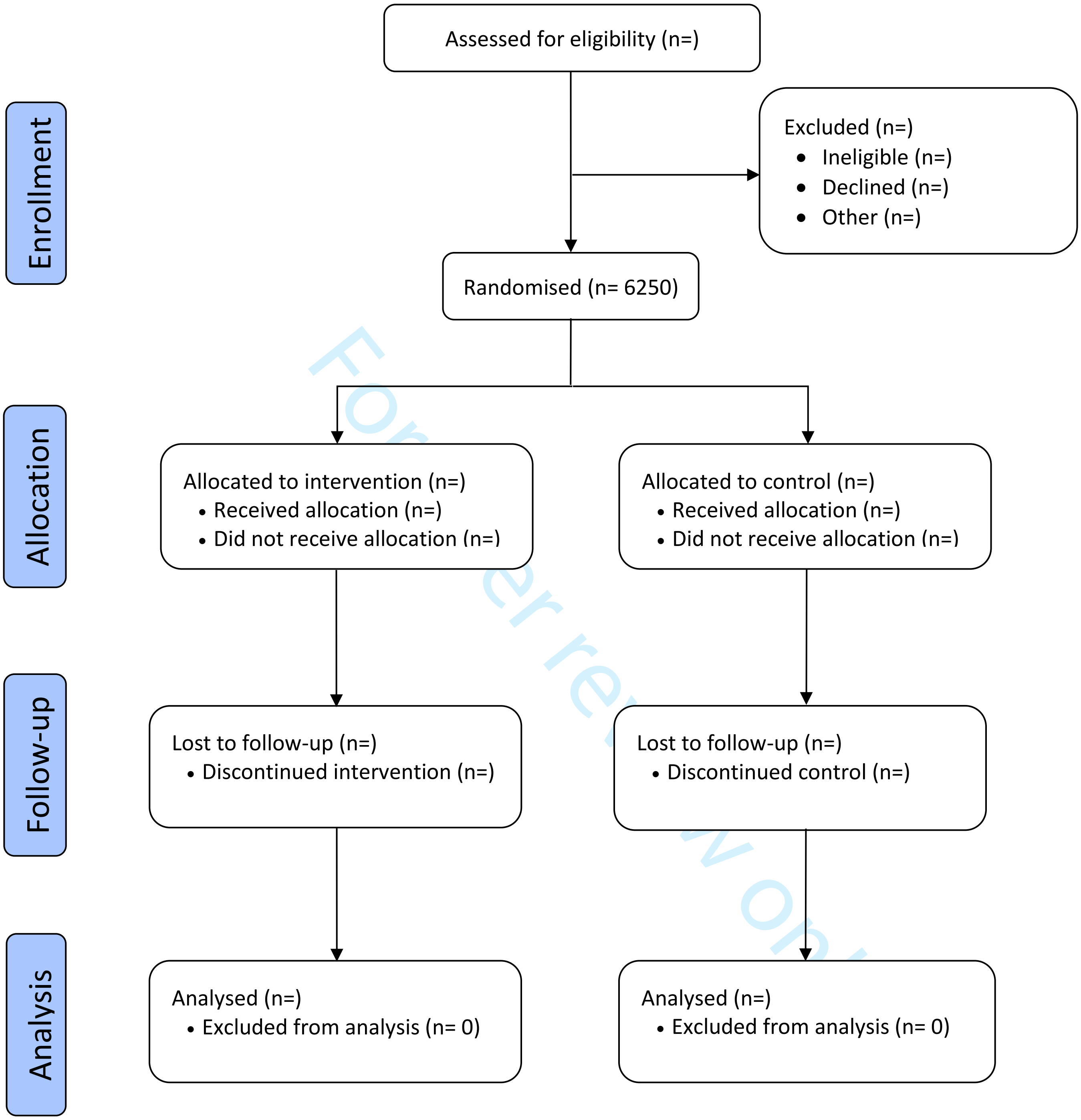
Enrollment
Assessed for eligibility (n=)
Excluded (n=)
Ineligible (n=)
Declined (n=)
Other (n=)
Randomised (n= 6250)
Allocation
Allocated to intervention (n=)
Received allocation (n=)
Did not receive allocation (n=)
Allocated to control (n=)
Received allocation (n=)
Did not receive allocation (n=)
Follow-up
Lost to follow-up (n=)
Discontinued intervention (n=)
Lost to follow-up (n=)
Discontinued control (n=)
Analysis
Analysed (n=)
Excluded from analysis (n= 0)
Analysed (n=)
Excluded from analysis (n= 0)

Figure 2. Schedule of enrolment, interventions, and assessments.

	STUDY PERIOD				
	**Enrolment**	**Allocation**	**Post-allocation**		**Close-out**
**TIMEPOINT****	**0**	**0**	***Wk 4***	***Wk 52***	***Wk 76***
**ENROLMENT:**					
**Eligibility screen**	X				
**Informed consent**	X				
**Allocation**		X			
**INTERVENTIONS:**					
safetxt text messages		◆——	———	——◆	
control text messages		◆——	———	——◆	
**ASSESSMENTS:**					
Socio-demographic characteristics	X				
Recent sexual behaviour	X		X	X	X
STI diagnosis	X				
Correct treatment of STI			X		
Partner notification			X		
Attitudes, self-efficacy, knowledge			X		
STI test (postal NAAT)				X	X
STI testing prior to sex with new partner				X	X
Number of text messages read				X	X
Anyone else read text messages				X	X
Experience of car accident				X	X
Experience of partner violence				X	X

*

For peer review only

A randomised controlled trial of a safer sex intervention delivered through mobile phone messaging

Local site logo

Participant's Information Sheet
safetxt: a randomised controlled trial of a safer sex intervention

We are inviting you to take part in a research study. Before you decide, it is important that you know why we are doing the study and what is involved. Please read the following information carefully.

What is the study?
This study is testing whether text messages providing information and tips to increase safer sex helps young people adopt safer sex behaviours.

Why have I been chosen?
You are aged 16-24 and have recently received a positive test result for Chlamydia, gonorrhoea or had a non specific urethritis diagnosis.

Do I have to take part?
No, it is up to you to decide whether to take part.

What will happen if I take part?
After you have had all your questions answered and have agreed to take part, we will ask you to complete a consent form. You can do this on our secure and confidential study website, by filling out a paper version, by text message or by email. We will then ask you to complete a short confidential questionnaire providing details about yourself.

After you have completed the questionnaire, **an automated computer system will put you into one of two groups by chance (randomly)**. One group will receive safer sex text messages for 1-2 weeks, then about 5 messages a month for 11 months. The other group will receive text messages asking you to confirm your contact details. These messages will not be about sexual health. This group will receive one message a month for 12 months.

We will ask you to complete the questionnaire again at 1 and 12 months after signing up. This is to see how things have been for you after joining the study. Also at 12 months we will ask you to provide a sample to test for Chlamydia and gonorrhoea. The testing kit will be sent to you by post. **Participating in the study involves returning one urine sample or self-taken swab to test for Chlamydia and gonorrhoea.**

Will you compensate me for the time this takes?
We will offer you £5 as compensation for your time at each follow up: for the 1 month questionnaire and the 12 month questionnaire. You will be compensated for your time for returning your 12 month sample.

We do not charge for text messages we send to you.

What are the alternatives?
You do not have to take part.

What are the possible disadvantages in taking part?
Completing the questionnaires and providing a sample will take up some of your time. It is possible that the messages we send could be read by someone else. If you are concerned about this, you can password-lock your phone and delete the messages after you read them.

For peer review only - http://bmjopen.bmj.com/site/about/guidelines.xhtml

What are the possible benefits of taking part?
We cannot promise that your health will benefit from taking part but you may find the messages helpful and you may learn about safer sex behaviours.

What happens when this study stops?
When the study stops, we will look at the data collected to see if the text messages helped increase safer sex behaviours.

What will happen if I don't want to carry on with the study?
You can stop receiving text messages by texting STOP or pause the text messages by texting PAUSE. You can withdraw at any time by letting the Clinical Trials Unit know (safetxt@LSHTM.ac.uk or freephone 0800 008 7197). You do not have to give a reason for wanting to withdraw.

What if there is a problem?
You can contact the Clinical Trials Unit if there is a problem. If you would like to make a formal complaint, contact Dr Caroline Free who will follow the complaints procedure.

Will my taking part in this study be kept confidential?
Yes. Your answers to the questionnaires will be stored anonymously and your contact details and Chlamydia and gonorrhoea test will be kept confidential. You will be allocated a unique study number when you enrol in the study. We will not inform your parents about your involvement in this research. If you were tested for sexually transmitted infections during the study, we will ask your clinic or GP for your test results. We will also ask for information on whether or not a partner was treated. To keep the messages confidential, password-lock your phone and delete messages after you read them.

Your records will be made available to people authorised to work on the study. They may also need to be made available to people for ensuring that the study is carried our correctly.

With your permission, we will ask your clinic or GP for your test results for sexually transmitted infections after you have finished participating in the study. This is to allow the study researchers to look into long term effects about your sexual health.

What will happen to the samples that I give?
You will post your sample directly to a laboratory to test for Chlamydia and gonorrhoea infection. The laboratory will destroy the samples after they have finished testing. You will be notified if the test shows that there is Chlamydia or gonorrhoea infection. The clinic where you signed up to the study will contact you to arrange treatment.

How long will your data be stored for?
Your data will be kept for 10 years after the trial is completed. However, in accordance with the Data Protection Act of 1998, any personal identifiable data will not be kept longer than necessary and will be deleted within 3 months after you have finished the study.

What will happen to the results of the research study?
The results will be published in a scientific journal so that other people know about it. If you would like a copy of the results please contact the Clinical Trials Unit.

If you want to receive the safer sex messages after your participation in the study, please let us know and we will send them to you.

If the results of the study show that the messages have helped, they will be made available to all young people.

Who is organising and funding the research?
The study is run by Dr Caroline Free (Chief Investigator) from the London School of Hygiene and Tropical Medicine, at the University of London. The UK National Institute for Health Research is funding the trial.

For peer review only - http://bmjopen.bmj.com/site/about/guidelines.xhtml

Who has reviewed the trial?

London Riverside NHS Medical Research Ethics Committee.

Thank you for taking the time to consider taking part.

If you would like further information please contact the local Research Nurse or Clinical Trials Unit (details below)

The team contact details:

INSERT LOCAL CONTACT DETAILS

If you have any questions about the trial, please email: safetxt@LSHTM.ac.uk or Freephone: 0800 008 7197

For peer review only - http://bmjopen.bmj.com/site/about/guidelines.xhtml

safetxt
A randomised controlled trial of a safer sex intervention delivered through mobile phone messaging

Hospital logo to be inserted

Consent Form

Chief Investigator: Dr Caroline Free

Name of local researcher: *(Please insert local details)*

Please **initial box**

I confirm that I have read and understand the information sheet [version 8, 16th April 2018] for the above study. I have had the opportunity to consider the information, ask questions and have had these answered satisfactorily. ☐

I understand that participation in this study is my choice and that I am free to withdraw at any time without giving any reason, without my medical care or legal rights being affected. ☐

I understand that I should not read or send text messages while driving a vehicle. ☐

I understand that relevant sections of data collected during the study may be looked at by the NHS Trust or LSHTM, where it is relevant to my taking part in this research. I give permission for these individuals to have access to my records. ☐

I agree to the study researchers letting my clinic know of any positive test results I receive so they can arrange treatment. ☐

I agree that my clinic/GP can provide the researchers with my test results for sexually transmitted infections. ☐

If I give my partner(s) details to my clinic, I agree to the researchers being told if they have been tested. ☐

I understand that all information I provide will remain confidential in accordance with the Data Protection Act of 1998. ☐

I understand that the information collected about me will be used to support other research in the future, and may be shared anonymously with other researchers. ☐

I agree to take part in the above study. ☐

Optional

After the study, I agree that my clinic/GP can provide the researchers with my sexually transmitted infection results. ☐

Name of Patient	Date and time	Signature
Name of researcher taking consent	Date and time	Signature

For peer review only - http://bmjopen.bmj.com/site/about/guidelines.xhtml

STANDARD PROTOCOL ITEMS: RECOMMENDATIONS FOR INTERVENTIONAL TRIALS

SPIRIT 2013 Checklist: Recommended items to address in a clinical trial protocol and related documents*

Section/item	Item No	Description	Addressed on page number
**Administrative information**			
Title	1	Descriptive title identifying the study design, population, interventions, and, if applicable, trial acronym	1
Trial registration	2a	Trial identifier and registry name. If not yet registered, name of intended registry	3
	2b	All items from the World Health Organization Trial Registration Data Set	3
Protocol version	3	Date and version identifier	3
Funding	4	Sources and types of financial, material, and other support	18
Roles and responsibilities	5a	Names, affiliations, and roles of protocol contributors	18
	5b	Name and contact information for the trial sponsor	19
	5c	Role of study sponsor and funders, if any, in study design; collection, management, analysis, and interpretation of data; writing of the report; and the decision to submit the report for publication, including whether they will have ultimate authority over any of these activities	19
	5d	Composition, roles, and responsibilities of the coordinating centre, steering committee, endpoint adjudication committee, data management team, and other individuals or groups overseeing the trial, if applicable (see Item 21a for data monitoring committee)	19

For peer review only

Introduction

Background and rationale	6a	Description of research question and justification for undertaking the trial, including summary of relevant studies (published and unpublished) examining benefits and harms for each intervention	5-6
	6b	Explanation for choice of comparators	8-9
Objectives	7	Specific objectives or hypotheses	6
Trial design	8	Description of trial design including type of trial (eg, parallel group, crossover, factorial, single group), allocation ratio, and framework (eg, superiority, equivalence, noninferiority, exploratory)	6

Methods: Participants, interventions, and outcomes

Study setting	9	Description of study settings (eg, community clinic, academic hospital) and list of countries where data will be collected. Reference to where list of study sites can be obtained	6
Eligibility criteria	10	Inclusion and exclusion criteria for participants. If applicable, eligibility criteria for study centres and individuals who will perform the interventions (eg, surgeons, psychotherapists)	6-7
Interventions	11a	Interventions for each group with sufficient detail to allow replication, including how and when they will be administered	8-9
	11b	Criteria for discontinuing or modifying allocated interventions for a given trial participant (eg, drug dose change in response to harms, participant request, or improving/worsening disease)	14
	11c	Strategies to improve adherence to intervention protocols, and any procedures for monitoring adherence (eg, drug tablet return, laboratory tests)	n/a
	11d	Relevant concomitant care and interventions that are permitted or prohibited during the trial	9
Outcomes	12	Primary, secondary, and other outcomes, including the specific measurement variable (eg, systolic blood pressure), analysis metric (eg, change from baseline, final value, time to event), method of aggregation (eg, median, proportion), and time point for each outcome. Explanation of the clinical relevance of chosen efficacy and harm outcomes is strongly recommended	9-10
Participant timeline	13	Time schedule of enrolment, interventions (including any run-ins and washouts), assessments, and visits for participants. A schematic diagram is highly recommended (see Figure)	Figure 2

For peer review only - http://bmjopen.bmj.com/site/about/guidelines.xhtml

Sample size	14	Estimated number of participants needed to achieve study objectives and how it was determined, including clinical and statistical assumptions supporting any sample size calculations	12
Recruitment	15	Strategies for achieving adequate participant enrolment to reach target sample size	7
**Methods: Assignment of interventions (for controlled trials)**			
Allocation:			
Sequence generation	16a	Method of generating the allocation sequence (eg, computer-generated random numbers), and list of any factors for stratification. To reduce predictability of a random sequence, details of any planned restriction (eg, blocking) should be provided in a separate document that is unavailable to those who enrol participants or assign interventions	7
Allocation concealment mechanism	16b	Mechanism of implementing the allocation sequence (eg, central telephone; sequentially numbered, opaque, sealed envelopes), describing any steps to conceal the sequence until interventions are assigned	7
Implementation	16c	Who will generate the allocation sequence, who will enrol participants, and who will assign participants to interventions	7
Blinding (masking)	17a	Who will be blinded after assignment to interventions (eg, trial participants, care providers, outcome assessors, data analysts), and how	7
	17b	If blinded, circumstances under which unblinding is permissible, and procedure for revealing a participant's allocated intervention during the trial	n/a
**Methods: Data collection, management, and analysis**			
Data collection methods	18a	Plans for assessment and collection of outcome, baseline, and other trial data, including any related processes to promote data quality (eg, duplicate measurements, training of assessors) and a description of study instruments (eg, questionnaires, laboratory tests) along with their reliability and validity, if known. Reference to where data collection forms can be found, if not in the protocol	11
	18b	Plans to promote participant retention and complete follow-up, including list of any outcome data to be collected for participants who discontinue or deviate from intervention protocols	11

For peer review only - http://bmjopen.bmj.com/site/about/guidelines.xhtml

Data management	19	Plans for data entry, coding, security, and storage, including any related processes to promote data quality (eg, double data entry; range checks for data values). Reference to where details of data management procedures can be found, if not in the protocol	11-12
Statistical methods	20a	Statistical methods for analysing primary and secondary outcomes. Reference to where other details of the statistical analysis plan can be found, if not in the protocol	12-14
	20b	Methods for any additional analyses (eg, subgroup and adjusted analyses)	13
	20c	Definition of analysis population relating to protocol non-adherence (eg, as randomised analysis), and any statistical methods to handle missing data (eg, multiple imputation)	13
**Methods: Monitoring**			
Data monitoring	21a	Composition of data monitoring committee (DMC); summary of its role and reporting structure; statement of whether it is independent from the sponsor and competing interests; and reference to where further details about its charter can be found, if not in the protocol. Alternatively, an explanation of why a DMC is not needed	14
	21b	Description of any interim analyses and stopping guidelines, including who will have access to these interim results and make the final decision to terminate the trial	14
Harms	22	Plans for collecting, assessing, reporting, and managing solicited and spontaneously reported adverse events and other unintended effects of trial interventions or trial conduct	14
Auditing	23	Frequency and procedures for auditing trial conduct, if any, and whether the process will be independent from investigators and the sponsor	14
**Ethics and dissemination**			
Research ethics approval	24	Plans for seeking research ethics committee/institutional review board (REC/IRB) approval	15
Protocol amendments	25	Plans for communicating important protocol modifications (eg, changes to eligibility criteria, outcomes, analyses) to relevant parties (eg, investigators, REC/IRBs, trial participants, trial registries, journals, regulators)	15

For peer review only - http://bmjopen.bmj.com/site/about/guidelines.xhtml

Consent or assent	26a	Who will obtain informed consent or assent from potential trial participants or authorised surrogates, and how (see Item 32)	7
	26b	Additional consent provisions for collection and use of participant data and biological specimens in ancillary studies, if applicable	n/a
Confidentiality	27	How personal information about potential and enrolled participants will be collected, shared, and maintained in order to protect confidentiality before, during, and after the trial	11-12
Declaration of interests	28	Financial and other competing interests for principal investigators for the overall trial and each study site	18
Access to data	29	Statement of who will have access to the final trial dataset, and disclosure of contractual agreements that limit such access for investigators	15
Ancillary and post-trial care	30	Provisions, if any, for ancillary and post-trial care, and for compensation to those who suffer harm from trial participation	n/a
Dissemination policy	31a	Plans for investigators and sponsor to communicate trial results to participants, healthcare professionals, the public, and other relevant groups (eg, via publication, reporting in results databases, or other data sharing arrangements), including any publication restrictions	15
	31b	Authorship eligibility guidelines and any intended use of professional writers	15
	31c	Plans, if any, for granting public access to the full protocol, participant-level dataset, and statistical code	n/a
**Appendices**			
Informed consent materials	32	Model consent form and other related documentation given to participants and authorised surrogates	Supplementary Files 1&2
Biological specimens	33	Plans for collection, laboratory evaluation, and storage of biological specimens for genetic or molecular analysis in the current trial and for future use in ancillary studies, if applicable	11

*It is strongly recommended that this checklist be read in conjunction with the SPIRIT 2013 Explanation & Elaboration for important clarification on the items. Amendments to the protocol should be tracked and dated. The SPIRIT checklist is copyrighted by the SPIRIT Group under the Creative Commons "Attribution-NonCommercial-NoDerivs 3.0 Unported" license.

For peer review only - http://bmjopen.bmj.com/site/about/guidelines.xhtml
